# Mixed Plantations Mitigate Negative Effects of Natural Forest Conversion on Soil Meso- and Micro-Faunal Communities in Zhangguangcailing Mountains, Northeast China

**DOI:** 10.3390/biology15141198

**Published:** 2026-07-20

**Authors:** Shuangjiao Ma, Yong Zhang, Kun Li, Qingcheng Wang, Donghai Cui, Zengwang Yao, Chuanrong Li, Yehan Tian

**Affiliations:** 1 Mountain Tai Forest Ecosystem Research Station of State Forestry Administration, Research Center for Forest Carbon Neutrality Engineering of Shandong Higher Education Institutions, Tai’an 271018, China; 2 College of Forestry, Southwest Forestry University, Kunming 650224, China; 3 College of Forestry, Northeast Forestry University, Harbin 150040, China

**Keywords:** forest conversion, secondary forest, plantation, soil meso- and micro-fauna, community structure

## Abstract

Soil meso- and micro-fauna are key components of forest soil ecosystems, but the effects of converting natural forests to monoculture or mixed plantations on their communities remain unclear. This study compared soil meso- and micro-fauna communities in monoculture (broad-leaved and coniferous) and mixed plantations converted from secondary forests in Northeast China. Results showed that monocultures significantly altered faunal community composition and reduced diversity, whereas mixed plantations maintained similar abundance and diversity to secondary forests. For instance, broadleaf monocultures exhibited a 15–23% lower Shannon–Wiener diversity index, while coniferous monocultures displayed a 28–35% reduction in soil faunal abundance. Notably, different taxonomic groups responded inconsistently to stand type conversion: Oribatida (58.8–72.5% of total individuals) varied significantly among stands (*p* < 0.001), whereas Collembola (18.8–31.4%) was more affected by season. The community composition was significantly correlated with understory plant diversity/coverage and soil C/N ratio, while α-diversity was mainly driven by total nitrogen. Our findings reveal that mixed plantations, instead of monocultures, are more effective for soil biodiversity conservation, which offers sustainable strategies for forest management.

## Simple Summary

The conversion from natural forests to plantations is a key global land-use change which exerts significant impacts on tree species composition and the soil environment. Soil meso- and micro-fauna are important drivers in the biogeochemical cycle of forest ecosystems, sensitive to environmental changes. However, research on how converting natural forests to plantations of varying tree species affects soil meso- and micro-faunal community structure remains limited, with no consistent findings to date. This study compared soil meso- and micro-faunal communities in monoculture and mixed plantations converted from secondary forests in Northeast China. Results showed that monocultures reduced faunal diversity and altered community composition, whereas mixed plantations maintained comparable abundance and diversity to natural forests. Understory vegetation and soil C/N ratio drove community composition variation, while soil total nitrogen determined α-diversity. These findings suggest that mixed plantations—rather than monocultures- effectively conserve soil biodiversity during forest conversion, supporting sustainable forest management strategies.

## 1. Introduction

Soil meso- and micro-fauna represent among the most diverse invertebrate groups in soil ecosystems [1] and contribute substantially to organic matter decomposition and nutrient turnover [2,3]. By consuming litter, ingesting microorganisms [4], or inducing trophic cascade effects [5,6], they not only regulate litter decomposition and soil nutrient and carbon cycling, but also influence the primary productivity of host plants by mediating the development of plant–fungus symbionts and regulating the dispersal of microbial propagules [7,8]. At the regional scale, soil meso- and micro-fauna are sensitive to changes in vegetation type [9], litter quantity and quality [10], and soil properties [11], and thus serve as important indicators of ecosystem health.

The conversion of natural forests to plantations constitutes a key global land-use change, exerting profound impacts on belowground ecosystems by altering vegetation composition, litter properties, and soil microhabitats [12,13]. Studies have shown that converting natural forests to monoculture plantations significantly reduces the abundance and diversity of soil meso- and micro-faunal communities [12]. However, other research has indicated that soil meso- and micro-faunal decomposers may exhibit preferences for specific litter types [9,14]. For instance, Wu et al. (2014) reported that deciduous broad-leaved forests are more conducive to the survival of Acari, while coniferous forests are more suitable for Collembola communities [15]. Therefore, no consistent conclusions have been drawn regarding the effects of converting natural forests to monoculture plantations on the functional groups community composition and quantitative characteristics of soil meso- and micro-fauna, as results may vary depending on the tree species composition of the stands. This is largely attributable to distinct litter quality and nutrient supply characteristics among different tree species, coupled with the divergent niche requirements of soil biota in terms of soil nutrients, root exudates, and microhabitat conditions, which jointly mediate the response patterns of soil faunal communities to forest conversion [16,17]. Exploring the response patterns of soil fauna to stand type conversion will help deepen the understanding of aboveground–belowground ecosystem interactions, and provide a scientific basis for the close-to-natural management of plantations, biodiversity conservation, and soil health improvement.

Compared with monocultures, mixed stands provide diversified habitats and litter types, which may be more conducive to the survival of soil meso- and micro-fauna [18,19]. Tree species mixing increases stand-scale habitat heterogeneity and supplies diverse litter and root-derived resources, which provide abundant ecological niches and food resources for soil biota, facilitate the coexistence of various soil faunal taxa, and elevate soil faunal community diversity [19]. Meanwhile, heterogeneous microhabitats and diversified resource inputs generated by mixed stands can buffer environmental fluctuation, reduce community temporal variability [20,21], and improve the structural stability of soil faunal communities. However, existing studies have indicated that mixed forests do not necessarily support more diverse soil meso- and micro-fauna than monocultures: the highest abundance and diversity often occur in monocultures of specific tree species within mixed forest systems [22,23], or no significant differences are observed between mixed and monoculture stands [24,25]. Only a limited number of studies have demonstrated that tree species mixing or increased litter diversity can enhance the diversity of soil meso- and micro-fauna [26,27]. Additionally, different faunal groups—such as Collembola [28] and Mesostigmata [24]—often respond differently to tree species mixing. Thus, it remains unclear whether converting natural forests to mixed-species plantations can mitigate the negative impacts on the community structure of soil meso- and micro-fauna compared with monocultures.

Besides altering food resources, stand type conversion can also influence soil faunal communities by modifying environmental conditions. Studies have shown that soil fauna are sensitive to changes in soil properties and vegetation cover [29,30], and variations in their abundance can be linked to differences in understory plant biomass and diversity [25], soil temperature and moisture [31], soil nutrient availability [12,32], and other factors. Moreover, different taxonomic groups of soil meso- and micro-fauna exhibit divergent responses to habitat alteration [33]. For instance, Collembola may be more responsive to understory vegetation or soil properties than Acari [15]. Nevertheless, the key environmental factors driving shifts in soil meso- and micro-faunal community structure following stand type conversion remain unclear. Accordingly, existing research has demonstrated pronounced seasonal variation in soil faunal community structure [1,34,35], reflecting seasonal changes in microclimatic conditions and resource availability. However, most existing studies only focus on a single season [12] and ignore how the interactive effects of season and forest type reshape the community structure of soil fauna. Therefore, examining seasonal dynamics in the community structure of soil meso- and micro-fauna across different stand types is essential for improving our understanding of the long-term effects of stand type conversion on these faunal communities.

We hypothesized that: (1) converting natural forests to plantations directly alters the community composition and diversity of soil meso- and micro-fauna, with such effects being weaker in mixed forests than in monocultures, and this pattern remains consistent across seasons; and (2) understory vegetation and soil properties may collectively act as environmental factors shaping the soil meso- and micro-faunal community.

At the Maoershan Research Forest Farm affiliated with Northeast Forestry University on the western slope of the Zhangguangcai Mountains, large areas of secondary forests (dominated by deciduous broad-leaved mixed forests) with similar site conditions have been converted to plantations. For this study, three plantation types—broad-leaved monoculture, coniferous monoculture, and coniferous-broadleaved mixed forest—established in 1987 at the Jianlagou Forest Cultivation Experimental Station were selected as research subjects, with adjacent retained secondary forests serving as the control. The aim was to explore the effects and underlying mechanisms of stand type conversion on the community structure of soil meso- and micro-fauna, thereby providing a scientific basis for forest ecosystem restoration and biodiversity conservation.

## 2. Materials and Methods

### 2.1. Study Site Description

This study was conducted at the Maoershan Research Forest Farm, affiliated with Northeast Forestry University, located in Heilongjiang Province, China (45°25′ N, 127°38′ E; average elevation: 300 m). The study area lies within a branch of the Changbai Mountains, featuring a landform dominated by low mountains and hills. It has a temperate continental monsoon climate, with an annual average temperature of 2.8 °C, and monthly average temperatures ranging from −19.6 °C (January) to 20.9 °C (July). The frost-free period lasts 120–140 days per year. Annual precipitation is approximately 600–800 mm, while annual evapotranspiration reaches around 1094 mm. The soil in the region is classified as Hap-Boric Luvisol: the 1–10 cm soil layer is loam, and the 10–20 cm layer is sandy loam.

The dominant vegetation consists of secondary forests, which developed from the cutover sites of ancient Korean pine-broadleaved mixed forests approximately a century ago. The main tree species include *Fraxinus mandshurica*, *Juglans mandshurica*, *Phellodendron amurense*, *Pinus koraiensis*, *Betula platyphylla*, *Quercus mongolica*, *Populus davidiana*, *Tilia amurensis*, *Ulmus pumila*, and *Acer mono* [36]. The primary understory shrubs are *Ribes mandshuricum*, *Corylus heterophylla*, and *Padus racemosa*, while the dominant understory herbaceous species include *Athyrium brevifrons*, *Brachybotrys paridiformis*, and *Urtica angustifolia*, among others.

**Figure 1:**
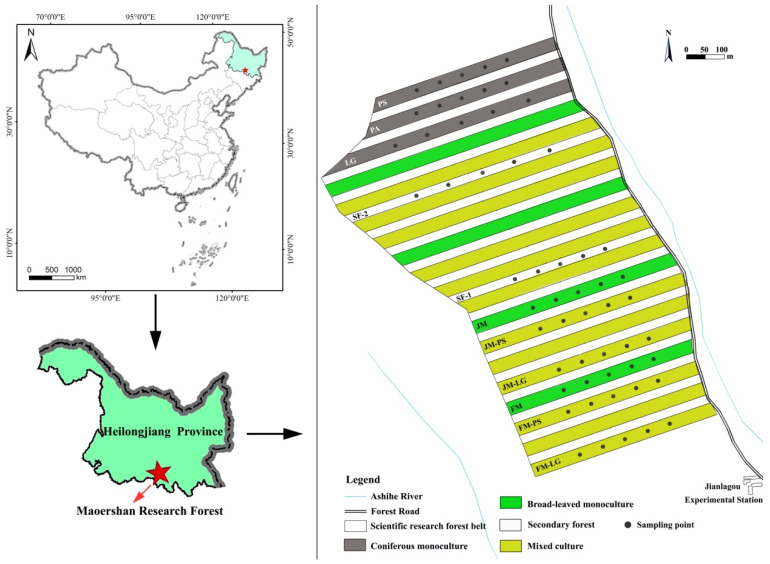
Location and distribution of the study area and the targeted stands in Maoershan Research Forest Farm, Northeast China. Note: FM, JM, LG, PS, PA, FM-LG, FM-PS, JM-LG, JM-PS for plantation forest stands composed of *Fraxinus mandshurica*, *Juglans mandshurica*, *Larix gmelinii*, *Pinus koraiensis*, *Picea koraiensis*, *Fraxinus mandshurica* and *Larix gmelinii*, *Fraxinus mandshurica* and *Pinus koraiensis*, *Juglans mandshurica* and *Larix gmelinii*, *Juglans mandshurica* and *Pinus koraiensis*, respectively. SF-1 and SF-2 for the second forest stands were mainly composed of *Fraxinus mandshurica*, *Betula platyphylla*, *Ulmus davidiana*, *Juglans mandshurica*, *Quercus mongolica*, *Acer mono*, *Tilia amurensis*.

The experimental stands were initially established in 1987. A large area of secondary forests with uniform site conditions was clear-cut in strips wider than 30 m, with secondary forest strips of the same width retained alternately. Subsequently, monoculture and mixed plantations were established with a planting spacing of 1.5 m × 2.0 m. In mixed stands, coniferous and broadleaved tree species were arranged alternately in contiguous blocks: five rows of conifers followed by three rows of broadleaves. Nine plantation stands with contrasting tree species compositions and two adjacent retained natural forest stands were selected for this study (Figure 1, Table 1). All stands were located on a uniform slope with a gradient ≤ 10°, under relatively consistent site conditions.

### 2.2. Experiment Layout and Sampling Design

In each target stand, five 1 m × 1 m plots were randomly established at intervals of at least 20 m. In mixed stands, sampling plots were positioned between adjacent rows of different tree species. Sampling was performed in summer (early August) and autumn (early October) 2020, as well as in spring (early June) 2021. A baseline survey of the target stands was carried out in early August 2020. Prior to sampling, soil moisture (Mois) and temperature (Tem) in the 0–10 cm soil layer were measured using a TDR device (TDR 300, Spectrum, Aurora, IL, USA) and a needle-type soil thermometer (PX-08H, PXTONG, Guangzhou, China), respectively. Litter thickness (OL/OF/OH), as well as the coverage, species composition, and abundance of shrubs and herbs, were recorded.

Soil meso- and micro-fauna samples were collected at the center of each sampling plot. Litter horizons (OL/OF/OH) within an area of 10 cm × 10 cm were sampled, together with two mineral soil layers using a 500 cm^3^ cutting ring (height: 6.4 cm, diameter: 10 cm). A total of 495 fauna samples (11 stands × 5 plots × 3 litter/soil horizons × 3 sampling seasons) were obtained from litter and mineral soils. All samples were placed in sealed plastic bags, transported immediately to the laboratory, and stored at 4 °C prior to extraction of soil meso- and micro-fauna.

During the first sampling campaign, three soil cores (0–10 cm depth) were collected from each plot using a soil auger (5 cm in diameter) and thoroughly mixed to form one composite sample. A total of 55 composite soil samples were obtained, stored in a cooler, and transported to the laboratory for subsequent processing. Soil samples were passed through a 2 mm sieve, and each sample was divided into two subsamples: one was stored at 4 °C for analysis of ammonium nitrogen and nitrate nitrogen, while the other was air-dried for further soil chemical analyses.

### 2.3. Identification of Soil Meso- and Micro-Fauna

Litter and soil meso- and micro-fauna were extracted using a modified Tullgren funnel apparatus for 48 h, and the extracted fauna were preserved in 75% ethanol. All individuals were counted under an Olympus stereomicroscope (SZX-ILLB2-200, Olympus Co., Tokyo, Japan). Most meso- and micro-fauna were identified to the order level, while better-known taxa such as Collembola, Coleoptera, and Araneae were identified to the family level under a Leica DM4000B binocular microscope (Leica Microsystems, Wetzlar, Germany) at 400× magnification. Faunal identification followed the taxonomic references of Yin (1998) [37] and Zhong (1990) [38]. To distinguish between adult and larval stages, individuals were counted separately, as they perform distinct ecological functions [39,40]. The identified fauna were assigned to different trophic groups, including predators, saprophages, omnivores, phytophages, and fungivores.

### 2.4. Soil Analysis

Soil physicochemical properties were determined following standard soil analytical protocols described in Chinese agricultural test guidelines [41]. Soil pH was measured in a 1:2.5 (soil:H_2_O, *w*/*v*) suspension after shaking for 2 h using a pH meter (MT-5000, Shanghai, China). Total phosphorus (TP) was determined colorimetrically using a UV-visible spectrophotometer (Agilent Technologies, Santa Clara, CA, USA) following wet digestion with a HClO_4_–H_2_SO_4_ mixture. Total potassium (TK) was analyzed by flame photometry after perchloric acid digestion. Available phosphorus (AP) was extracted with 0.05 mol/L hydrochloric acid—0.025 mol/L sulfuric acid and measured colorimetrically using the molybdenum–antimony ascorbic acid method with a UV-visible spectrophotometer. Available potassium (AK) was extracted with ammonium acetate and determined by flame photometry. Soil total carbon (TC) and total nitrogen (TN) were analyzed via dry combustion using a Vario MACRO elemental analyzer (Elementar Co., Ltd., Langenselbold, Germany) [42]. Ammonium nitrogen (NH_4_^+^-N) and nitrate nitrogen (NO_3_^−^-N) were quantified using a Bran-Luebbe Auto Analyzer 3 flow injection analyzer (Bran+Luebbe, Norderstedt, Germany) [43].

### 2.5. Community Structure Indexes

Data on soil meso- and micro-fauna across the three layers (one litter layer and two mineral soil horizons) were combined for statistical analysis. Mean abundance, taxon richness, Shannon–Wiener diversity index, and Pielou evenness index of soil meso- and micro-fauna, as well as the abundances of Oribatida, Collembola, Mesostigmata, and Prostigmata, were calculated separately for each sampling season and stand type. Shannon–Wiener diversity (H) [44] and Pielou evenness (E) [45] were computed based on the abundance and taxonomic richness of soil meso- and micro-fauna. Mean species richness, coverage, Shannon–Wiener diversity index (H), and Simpson dominance index (D) [46] of understory vegetation (shrubs and herbs) in August were also calculated.

### 2.6. Statistical Analyses

Shapiro–Wilk and Levene tests were conducted to test data normality and homogeneity of variances, respectively. To meet the assumptions of parametric statistical models, datasets describing soil meso- and micro-faunal abundance, taxonomic richness, Shannon–Wiener index and Pielou evenness index were natural log (ln) transformed prior to all statistical analyses. One-way analysis of variance (ANOVA) was used to examine variations in soil meso- and micro-faunal abundance, taxonomic richness, Shannon–Wiener diversity index, and Pielou evenness index among different stand types and sampling seasons. Additionally, one-way ANOVA was applied to analyze the effects of stand type on environmental variables (including understory vegetation characteristics and soil physicochemical properties) in August. When significant differences were detected, multiple comparisons of means were conducted using the least significant difference (LSD) test at a significance level of *p* < 0.05. Two-way ANOVA was employed to evaluate the impacts of stand type, sampling season, and their interaction on the abundance, taxonomic richness, Shannon–Wiener diversity index, and Pielou evenness index of soil meso- and micro-fauna, as well as the abundances of Oribatida, Collembola, Mesostigmata, and Prostigmata.

Non-metric multidimensional scaling (NMDS) was performed using the vegan package [47] based on the Bray–Curtis similarity coefficient, to investigate variations in soil meso- and micro-faunal community structure among forest stand types in June, August, and October. The reliability of the ordination was evaluated using stress values, and dominant soil fauna taxa were fitted to the ordination to identify key drivers of community dissimilarity.

Correlation analysis and Mantel test were performed between the soil meso- and micro-faunal community structure data matrix and the environmental factor matrix using the LinkET package [48]. The LinkET package was employed to generate a combined correlation analysis diagram, which simultaneously visualized the correlations among environmental factors and the relationships between these faunal community structures and ecological factors via an integrated correlation heatmap and network diagram. The soil meso- and micro-faunal community structure data included alpha-diversity indices (Shannon–Wiener diversity index, Pielou evenness index), community composition data of soil meso- and micro-fauna, and abundance data of Acari and Collembola in August. Environmental factors encompassed understory vegetation characteristics and soil physicochemical properties. Redundancy analysis (RDA) was conducted using the vegan package [47] to evaluate the effects of environmental variables on soil meso- and micro-faunal taxa. All statistical analyses were implemented in R ver. 3.6.1 for Windows [49].

## 3. Results

### 3.1. Community Composition of Soil Meso- and Micro-Fauna

A total of 164,346 individuals, representing 86 taxa belonging to three phyla, 11 classes, and 25 orders, were identified. Oribatida and Onychiuridae were the dominant groups, accounting for 52.5% and 10.8% of the total individuals, respectively. The common groups included Isotomidae (9.6%), Mesostigmata (8.4%), Diptera larvae (5.6%), Prostigmata (3.7%), Hypogastruridae (3.6%), and Enchytraeidae (1.4%) (Table S1). All remaining taxa were classified as rare groups, collectively comprising 4.3% of the total individuals (Table S1).

**Figure 2:**
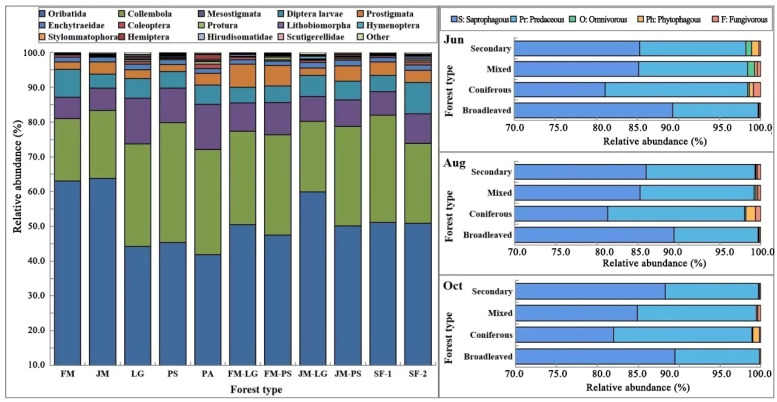
Taxonomic composition (**left panel**) and trophic group structure (**right panel**) of soil meso- and micro-fauna across different stand types.

The taxonomic composition of soil meso- and micro-fauna differed among forest stand types (Figure 2). Acari (including Oribatida, Prostigmata, and Mesostigmata) exhibited clear dominance in all stands, with the highest relative abundance in BL (72.5%) and the lowest in CF (58.8%). Collembola showed the opposite pattern: their relative abundance was lowest in BL (18.8%) and highest in CF (31.4%). Mixed stands (MIX) and natural secondary forest (SF) displayed intermediate proportions of Acari (61.7–69.1%) and Collembola (20.4–30.8%) (Figure 2). Across all sampling seasons, following the conversion of natural forests, the relative proportion of saprophagous faunal groups decreased significantly in CF stands, while the proportion of predatory groups increased. In contrast, the opposite trend was observed in BL stands. The trophic group structure showed little difference between MIX and SF (Figure 2).

**Figure 3:**
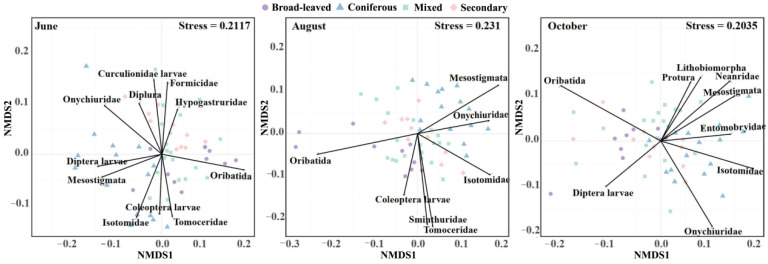
Non-metric multidimensional scaling ordination of soil meso- and micro-fauna across different stand types.

NMDS ordination based on Bray–Curtis distances revealed stress values of 0.2117, 0.231, and 0.2035 for soil meso- and micro-faunal communities in June, August, and October, respectively (Figure 3), all within the acceptable range of 0.2–0.3. The CF samples were relatively clustered across all three months and clearly separated from those of other stand types, particularly BL forests. The distribution of coniferous forest plots was strongly aligned with the directions of Onychiuridae and Isotomidae. The BL forest samples were mostly concentrated in distinct regions of the ordination space and closely associated with the vector direction of Oribatida in all months. The MIX forest samples were relatively scattered, with partial overlap with other stand types, and their distribution corresponded to the directions of taxa including Mesostigmata and Tomoceridae. The SF samples overlapped substantially with mixed forests across all three months, and their distribution was generally consistent with the vector directions of Formicidae and Diplura in June (Figure 3).

### 3.2. The Abundance and Diversity of Soil Meso- and Micro-Fauna

**Figure 4:**
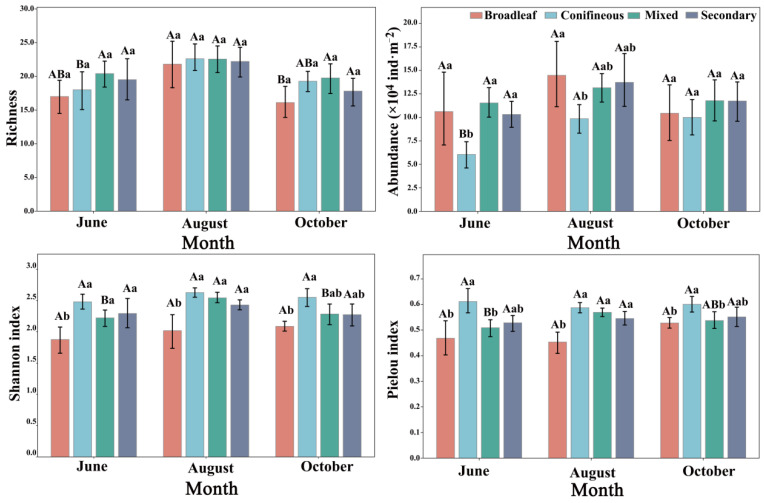
Variations of taxonomic richness, abundance, Shannon–Wiener and Pielou index of the soil meso- and micro-fauna community across different stand types. Uppercase letters on the bars indicate significant differences within seasons at the *p* < 0.05 level, while lowercase letters on the bars indicate differences within stands at the *p* < 0.05 level.

Taxonomic richness of soil meso- and micro-fauna was strongly affected by sampling season but independent of stand type (Figure 4, Table 2). In BL stands, soil meso- and micro-faunal richness was significantly higher in August than in October (*p* < 0.05), whereas in CF stands, richness was significantly greater in August than in June (*p* < 0.05). No significant seasonal variation in taxonomic richness was observed in SF and MIX stands (Figure 4). Stand type exerted a significant effect on the abundance of soil meso- and micro-fauna (Figure 4, Table 2). Abundance in CF stands was significantly lower than in the other stand types in June and August.

The Shannon–Wiener diversity index of soil meso- and micro-fauna was significantly affected by stand type and sampling season (Figure 4, Table 2). The Shannon–Wiener index in BL stands was the lowest among all stand types (*p* < 0.05). In MIX stands, the Shannon–Wiener index of soil meso- and micro-fauna was significantly higher in August than in other seasons (*p* < 0.05), whereas no significant seasonal differences were detected in the other stand types (Figure 4). The Pielou evenness index was also significantly influenced by stand type (*p* < 0.05; Table 2), with the highest value observed in CF and the lowest in BL (Figure 4).

**Figure 5:**
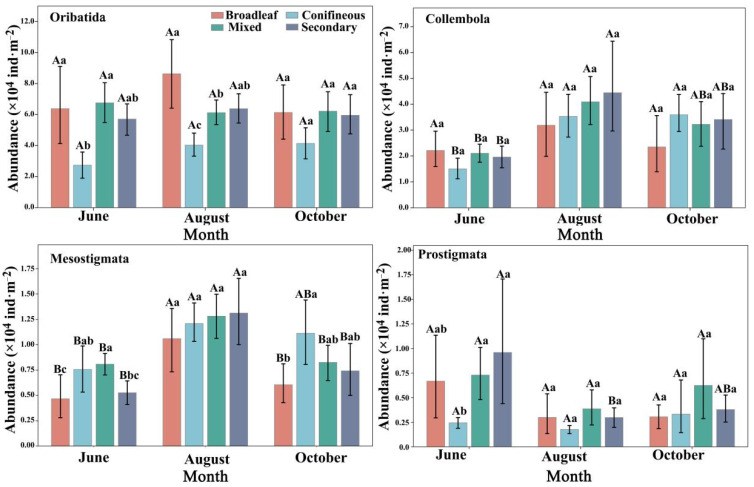
Variations of the abundance of Oribatida, Collembola, Mesostigmata, and Prostigmata across different stand types. Uppercase letters on the bars indicate significant differences within seasons at the *p* < 0.05 level, while lowercase letters on the bars indicate differences within stands at the *p* < 0.05 level.

The abundance of Oribatida was significantly affected by stand type (*p* < 0.05), whereas Collembola abundance was only influenced by sampling season (*p* < 0.05) (Table 3, Figure 5). The abundance of both Mesostigmata and Prostigmata was significantly affected by stand type and sampling season (*p* < 0.05) (Table 3, Figure 5). Abundances of Oribatida and Prostigmata were lower in CF than in other stand types, while Mesostigmata abundance was lowest in BL (Figure 5). In addition, Mesostigmata reached its highest abundance in August, whereas Prostigmata abundance was significantly higher in June than in other sampling seasons. Collembola abundance was significantly greater in August than in June across all stand types except BL (*p* < 0.05) (Figure 5).

### 3.3. Relationships Between Environmental Factors and Community Structure of Soil Meso- and Micro-Fauna

The Simpson index of understory vegetation, litter thickness (LT), soil temperature (Tem), and soil moisture (Mois) differed significantly among stand types (*p* < 0.05; Table 4 and Table S2). The Simpson index of understory vegetation was significantly higher in SF than in CF (*p* < 0.05; Table 4). Litter thickness was greatest and soil temperature lowest in CF (*p* < 0.05). Soil moisture in BL was significantly higher than in CF and MIX (*p* < 0.05), but did not differ significantly from that in SF (Table 4).

With the exception of total carbon (TC), total potassium (TK), all other soil chemical properties differed significantly among stand types (Table 5 and Table S3). Soil pH was significantly lower in plantation forests than in SF (*p* < 0.05), whereas SF exhibited the highest contents of total phosphorus (TP), available phosphorus (AP) and available potassium (AK). BL stands had the highest total nitrogen (TN) content and the lowest C/N ratio. Furthermore, most soil chemical properties in MIX stands were higher than those in CF. The lowest soil NO_3_^−^ concentration was observed in BL, while the lowest NH_4_^+^ concentration occurred in CF (Table 5).

**Figure 6:**
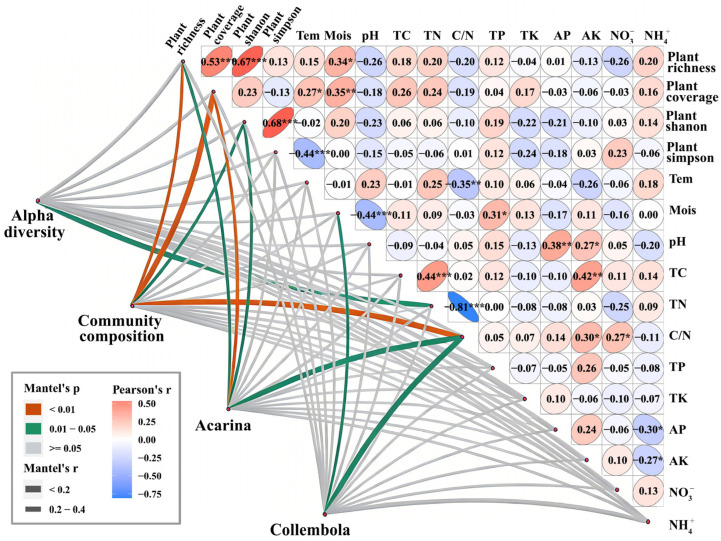
Correlation between mesofauna community structure and environmental factors across different stand types. The heat map shows the correlation between environmental factors. Colours indicate correlation types. The value displayed in the heat map grid denotes Pearson’s correlation coefficient for pairwise environmental factors comparisons. The network diagram shows the correlation between the soil meso- and micro-fauna data matrix and the environmental factor matrix. Edge width of the lines corresponds to the absolute value of the correlation coefficient. The color of the connecting line indicates the significance value. *, **, and *** indicate significant differences at the *p* < 0.05 level, *p* < 0.01 level, and *p* < 0.001 level, respectively.

**Figure 7:**
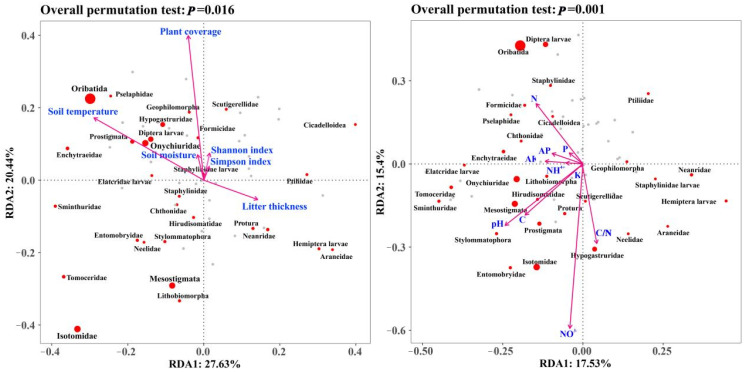
Redundancy analysis (RDA) between understory plants characteristics, soil moisture, soil temperature, liter thickness, and soil meso- and micro-fauna (**left**), and between soil chemical attributes and soil meso- and micro-fauna (**right**). Red circles are response variables (mesofauna), while vectors are explanatory variables (environmental factors).

The diversity of soil meso- and micro-fauna was influenced by multiple environmental factors (Figure 6). The alpha diversity of soil meso- and micro-fauna was only correlated with soil total nitrogen (TN). The community composition of soil meso- and micro-fauna varied significantly with understory plant diversity, understory coverage, and soil C/N ratio (*p* < 0.05), as did the abundance of Acari. The abundance of Collembola was significantly correlated with soil moisture (Mois) and C/N ratio (Figure 6). Redundancy analysis (RDA) illustrating the relationships between soil meso- and micro-faunal taxa and environmental variables showed that the first two axes explained 48% of the variation associated with understory plant characteristics and 33% of the variation related to soil chemical properties, respectively. The abundance of Oribatida and Diptera larvae were associated with higher soil temperature, greater understory coverage, and thinner litter thickness; and their abundance increased with increasing TN content and decreased with increasing soil C/N ratio, and NO_3_^−^ concentration. The abundance of Isotomidae decreased with increasing understory coverage but increased with elevated soil pH and NO_3_^−^ concentration. Onychiuridae abundance was positively correlated with soil temperature and soil total carbon (TC) content. Mesostigmata abundance was linked to lower understory plant coverage but higher soil pH and total carbon (TC) content. Prostigmata exhibited a weak correlation with understory plant diversity, but was similarly positively associated with soil total carbon (TC) content. (Figure 7, Table S4).

## 4. Discussion

### 4.1. Effects of Forest Conversion on Community Composition of Soil Meso- and Micro-Fauna

In this study, conversion from secondary forests to monocultures significantly altered the community composition and diversity of soil meso- and micro-fauna, whereas conversion to mixed forests showed no significant effects across all sampling seasons. Thus, increasing tree species diversity during stand conversion exerts a buffering effect on soil meso- and micro-faunal community structure and facilitates its restoration. Our findings are consistent with previous soil fauna research, which has demonstrated that the α- and β-diversity of soil fauna decline markedly after tropical rainforests are converted to rubber plantations [12]. Nevertheless, other studies have indicated that stand type exerts negligible influence on soil faunal community composition [15,32]. The present study further demonstrates that the effects of forest conversion on soil faunal community structure depend on the tree species composition and diversity of post-conversion stands.

Previous studies have indicated that various factors, such as litter chemical composition [50] and soil C/N ratio [28], influence the community structure of soil meso- and micro-fauna. Additionally, understory vegetation modifies microhabitats and regulates soil biodiversity by affecting the quantity, chemical quality, and diversity of food inputs into the soil food web [10,25]. Our results showed that soil C/N ratio and understory plant characteristics played crucial roles in shaping the composition of soil meso- and micro-fauna (Figure 6). Differences in litter traits and root exudates across stands cause variable soil C/N ratios [51]. These changes modify soil nutrition and microbial biomass—the main food of soil meso- and micro-fauna [52,53]—and further drive community differentiation. The similar understory characteristics between mixed and secondary forests likely account for their comparable soil faunal communities. Coniferous monocultures exhibited distinct community compositions compared to other stand types, particularly broadleaf monocultures (Figure 3). Coniferous stands were dominated by Onychiuridae and Isotomidae (Collembola), while broadleaf stands were characterized by Oribatida as the core group, highlighting the importance of tree species traits. Variations in litter quantity and quality among different stands may ultimately filter distinct faunal assemblages by modifying food resources and habitat conditions [54].

Acari and Collembola represent the most abundant taxa of soil meso- and micro-fauna in forest soils and are widely used as effective indicators in biodiversity assessment [55]. In the present study, forest conversion did not alter the dominant position of Acari and Collembola (Figure 2). Previous studies have suggested that a higher abundance of Acari relative to Collembola reflects, to some extent, better soil quality and greater habitat stability in forest ecosystems [56]. The relative abundance of Oribatida increased significantly following conversion from natural forest to broadleaf plantation, decreased markedly in coniferous stands, and changed only slightly in mixed stands (Figure 2, Table S1). These results indicate that forest conversion reduced soil quality and stability in coniferous stands, whereas mixed tree species composition mitigated this trend to a certain degree. Oribatida remained dominant across all stand types, which is inconsistent with the findings of Wu et al. (2014) [15], who reported higher Acari abundance than Collembola in broadleaf stands but the opposite pattern in coniferous stands in the Hengduan Mountains. This discrepancy may be attributed to differences in climate, site conditions, and tree species characteristics among study regions [55,57]. All plantations in this study were converted from broadleaved natural forests, and this shared origin likely contributed to the consistent dominance of Oribatida.

In addition, the conversion of natural forests to coniferous monocultures led to a pronounced decrease in the proportion of saprophagous groups and a distinct increase in predaceous groups, whereas conversion to broadleaved monocultures showed the opposite trend. Broadleaf litter is characterized by high nitrogen content and a low lignin-to-nitrogen ratio, making it readily decomposable by microorganisms, thereby supporting a rich and diverse community of saprophagous (detritivorous) soil fauna, including abundant collembolans and mites that feed on bacteria, fungi, and detritus. In contrast, coniferous litter is rich in recalcitrant lignin and tannins with a high C/N ratio, which limits microbial activity and subsequently suppresses the abundance and diversity of saprophagous functional groups that rely on microorganisms and detritus as food resources [58]. Meanwhile, the microenvironment under coniferous stands tends to be homogenized and more stressful (e.g., low pH, low light availability) [18], further eliminating decomposition-sensitive faunal groups. Against this background, although the absolute abundance of predaceous soil fauna may not increase significantly, their relative proportion in community composition relatively increases because their prey (mainly saprophages) decline more rapidly. This phenomenon highlights the role of forest-type conversion in reshaping the functional structure of soil food webs from the top down by regulating litter quality and microhabitat conditions.

Notably, the effects of forest conversion on the community composition of soil meso- and micro-fauna are dependent on the target taxa and taxonomic resolution [59]. Future research should place greater emphasis on species-level differences in community composition. In summary, our hypothesis was supported: natural forest conversion directly alters the community composition of soil meso- and micro-fauna, with weaker effects observed in mixed stands than in monoculture plantations.

### 4.2. Effects of Forest Conversion on Community Diversity of Soil Meso- and Micro-Fauna

The abundance and diversity indices of soil meso- and micro-fauna were all affected by stand type (Figure 4, Table 2). Additionally, the conversion from secondary forests to coniferous monocultures significantly reduced their abundance in June. Evidence indicates that low soil nutrient content may exert a negative impact on soil fauna [15]; thus, the relatively low soil total nitrogen content could explain the lower abundance observed in coniferous stands (Table 5). Contrary to some previous studies, the thicker litter layer in coniferous monocultures may provide more food resources and diverse ecological niches for soil invertebrates, resulting in relatively high abundance [24,60]. Evidently, the abundance of soil meso- and micro-fauna in coniferous monocultures is jointly regulated by litter properties and soil characteristics.

Tree species mixing enhances habitat heterogeneity and enriches food resources, which often contributes to higher biodiversity [19]. However, some studies have reported that mixed stands do not necessarily improve soil faunal diversity [25,61]. In the present study, the diversity indices of soil meso- and micro-fauna in secondary forests and mixed forests were significantly higher than those in broadleaf monocultures, but showed no significant difference from those in coniferous monocultures. Although broadleaf monocultures provided high-quality food resources that supported large populations of Oribatida, their thin litter layer, relatively exposed soil environment, and limited food types may have reduced the diversity of soil meso- and micro-fauna. In contrast, the thicker litter layer in coniferous monocultures may offer niche space for more taxonomic groups [24], thereby promoting faunal diversification. This result is consistent with the findings of Meriç and Ender (2018) [62], who reported that the Shannon–Wiener index of soil microarthropods in Scots pine stands was 9.6% higher than that in natural oak stands. Therefore, whether converting secondary forests to mixed forests results in higher soil faunal abundance or diversity compared to monocultures depends on the tree species composition and specific stand characteristics. In addition, differences in the seasonal dynamics of various plant communities may lead to changes in microclimate, while resource availability and soil properties among different habitats directly or indirectly affect soil meso- and micro-faunal communities [50]. In this study, the taxon richness of soil meso- and micro-fauna in monocultures and the diversity index in mixed plantations showed obvious seasonal dynamics, with higher values in summer than in spring and autumn. By contrast, these indices showed no significant differences among the three seasons in secondary forests, suggesting that forest conversion affects the seasonal stability of soil faunal communities.

However, the two-way ANOVA revealed non-significant interactive effects between stand type and sampling season on all soil faunal community metrics (Table 2 and Table 3). This indicates that seasonal variation does not alter the overall response pattern of soil meso- and micro-fauna communities to forest conversion. Pure plantations (broadleaf and coniferous monocultures) consistently reduced soil faunal diversity and abundance relative to secondary forests and mixed plantations across all sampling seasons. Even though soil temperature, moisture, litter input, and understory vegetation obviously fluctuate over seasons, the filtering effect of stand structure on soil fauna communities remains stable throughout the year. Mixed plantations can sustain favorable microhabitat conditions (e.g., diversified litter resources, moderate soil C/N ratio and complete understory cover) in every season, which continuously buffer the negative impacts of forest transformation on soil fauna (Figure 4 and Figure 5). This finding aligns with previous studies reporting that mixed plantations sustain higher biodiversity and strengthen the disturbance resistance of ecosystems [20,63]. In contrast, monocultures maintain persistent adverse soil environments regardless of seasonal changes, including single litter quality, simpler understory vegetation, and unbalanced soil nutrient status (Table 4 and Table 5). Such consistent stand-specific environmental differences dominate faunal community variation, masking potential seasonal divergent responses among stand types and ultimately leading to a non-significant stand type × season interaction. This further validates Hypothesis (1), demonstrating that the conversion of secondary forests to mixed conifer–broadleaf plantations exerts weaker impacts on soil meso- and micro-faunal communities than conversion to monocultures, with this consistent pattern maintained across all sampling seasons.

### 4.3. Effects of Forest Conversion on Dominant Taxa of Soil Meso- and Micro-Fauna

Studies have shown that different groups of soil meso- and micro-fauna respond distinctively to forest disturbances [51,64]. For instance, Oribatida are regarded as K-strategists and generally exhibit weak responses to habitat changes [65,66], whereas Collembola are r-strategists that display rapid responses and quick recovery under environmental alterations [67]. However, the present study found that the abundance of Oribatida was significantly affected by forest conversion, while that of Collembola was not (Table 3). Based on stable isotope analysis, Oribatida span four trophic levels, including lichen feeders, fungal feeders, primary and secondary decomposers, and predators [68]. Our results indicate that differences in soil temperature, understory plant richness, and soil total nitrogen content among forest stands were the main drivers of variations in Oribatida abundance (Figure 7). Therefore, forest conversion may alter Oribatida abundance through bottom-up control by modifying the soil environment and food resources [69].

Collembola are closely associated with vegetation types [70] and act as important drivers of soil mineralization and nutrient availability [71]. In the present study, no significant differences in Collembola abundance were observed among different stand types (Figure 5), suggesting that Collembola communities had largely recovered 33 years following forest conversion. Our findings are consistent with previous studies reporting that Collembola abundance is not affected by stand type, structure, or corresponding habitats [23,51]. Some studies have noted that Collembola abundance is closely related to understory vegetation biomass or composition rather than soil properties [25]. However, our results indicated that Collembola abundance was unrelated to understory plant characteristics and was primarily influenced by soil moisture and C/N ratio (Figure 6), which implies that the dominant drivers of Collembola communities vary across study regions.

Mesostigmata and Prostigmata are predatory groups of soil meso- and micro-fauna [72] that prey on decomposer communities and are considered important regulators of decomposition processes [60]. Previous studies have reported that predatory taxa such as Mesostigmata exhibited significantly higher abundance in coniferous forests than in other stand types [24]. The present study similarly recorded higher Mesostigmata abundance in coniferous forests and coniferous-broadleaved mixed forests than in other stand types (Figure 5), further supporting the notion that the introduction of coniferous species during forest conversion can help enrich predatory faunal groups and maintain the diversity of soil food chains. In this study, Mesostigmata abundance was negatively correlated with understory plant cover and diversity, but positively correlated with soil pH (Figure 7), suggesting that dense and diverse understory vegetation may inhibit the predatory activity of this faunal group. The conversion from secondary forests to coniferous monocultures distinctly reduced understory plant richness and diversity (Table 4). Collectively, our second hypothesis—that understory vegetation and soil properties act as direct drivers of shifts in soil meso- and micro-faunal community structure—was also supported by the results.

Studies have documented seasonal dynamics in forest soil faunal communities in both high-latitude regions [15] and tropical areas [73]. Different faunal taxa also vary in their responses to climate change; for example, Collembola are highly sensitive to climatic fluctuations [55], whereas some soil mites exhibit stronger responses to temporal variation than to spatial variation [74]. The present study similarly found that taxon richness, diversity, and the abundance of dominant groups such as Collembola were significantly influenced by sampling season. Accordingly, multi-season surveys that incorporate seasonal variation in soil meso- and micro-faunal communities are essential for comprehensively assessing the impacts of forest conversion on soil faunal community structure.

## 5. Conclusions

The effects of conversion of natural forest into plantations on the composition of soil meso- and micro-fauna varied with tree diversity, with the significant differences between monocultures and SF, rather than between MIX and SF. Furthermore, broad-leaved monocultures tended to host more saprophagous taxa, while the establishment of coniferous monocultures could increase the proportion of predatory taxa. Mixed cultures exhibited functional group structures similar to those of secondary forests. Significant differences of α-diversity were observed between monoculture plantations and secondary forests (SF), but not between mixed forests (MIX) and secondary forests. In short, increasing tree species diversity in plantations established via natural forest conversion was conducive to the restoration and stability of soil meso- and micro-faunal community structure. Overall, forest conversion mainly affects the community composition of soil meso- and micro-fauna by altering understory plant diversity, vegetation coverage as well as soil C/N ratio, and regulated α-diversity mainly via changing soil total nitrogen content. Our findings provide valuable insights into the mechanisms underlying the effects of stand type conversion on the community structure of soil meso- and micro-fauna.

## Figures and Tables

**Table 1:** Tree species composition and stand characteristics of different stand types.

Stand Type	Stand Type Code	Tree Species Composition	Stand Code	Canopy Closure	DBH (cm)	Height (m)
Broad-leaved monoculture	BL	*F. mandshurica*	FM	0.9	13.70 ± 0.80	15.86 ± 0.61
		*J. mandshurica*	JM	0.8	18.65 ± 0.72	18.62 ± 0.59
Coniferous monoculture	CF	*L. gmelinii*	LG	0.8	20.51 ± 0.62	18.91 ± 0.37
		*P. koraiensis*	PS	0.9	15.15 ± 0.52	12.99 ± 0.35
		*P. koraiensis*	PA	0.9	16.05 ± 0.72	13.31 ± 0.43
Mixed culture	MIX	*F. mandschurica*	FM-LG	0.8	16.71 ± 0.73	20.53 ± 0.71
		*L. gmelinii*				
		*F. mandshurica*	FM-PS	0.9	14.03 ± 0.63	13.26 ± 0.39
		*P. koraiensis*				
		*J. mandshurica*	JM-LG	0.9	19.42 ± 0.72	21.11 ± 0.64
		*L. gmelinii*				
		*J. mandshurica*	JM-PS	0.9	15.92 ± 0.74	15.17 ± 0.81
		*P. koraiensis*				
Secondary forest	SF	*F. mandshurica*, *B. platyphylla*, *U. davidiana*, *J. mandshurica*, *A. mono*, *T. amurensis*	SF-1	0.9	24.73 ± 1.29	17.54 ± 0.56
			SF-2	0.8	21.83 ± 1.24	18.74 ± 0.70

Note: DBH: Average diameter at breast height of the stand trees; Height: Average tree height.

**Table 2:** Two-way ANOVA results for the effects of stand type, sampling season and their interaction on the taxonomic richness, abundance, Shannon–Wiener and Pielou index of the soil meso- and micro-fauna community across different stand types.

Source	df	Richness		Total Abundance		Shannon–Wiener		Pielou	
		*F*	*p*	*F*	*p*	*F*	*p*	*F*	*p*
Sampling season	2	11.795	0.000 ***	2.053	0.132	5.071	0.007 **	1.668	0.192
Stand type	3	2.161	0.095	11.042	0.000 ***	21.531	0.000 ***	17.65	0.000 ***
Stand type × Sampling season	6	0.536	0.78	1.267	0.276	0.921	0.482	2.083	0.058

Note: ** Significant at the *p* < 0.01 level; *** Significant at the *p* < 0.001 level.

**Table 3:** Two-way ANOVA results for the effects of stand type, sampling season, and their interaction on the abundance of Oribatida, Collembola, Mesostigmata, and Prostigmata across different stand types.

Source	df	Oribatida		Collembola		Mesostigmata		Prostigmata	
		*F*	*p*	*F*	*p*	*F*	*p*	*F*	*p*
Sampling season	2	1.669	0.192	15.082	0.000 ***	3.553	0.016 *	2.868	0.038 *
Stand type	3	13.999	0.001 **	1.032	0.38	22.375	0.000 ***	4.734	0.010 *
Stand type × Sampling season	6	1.284	0.268	0.86	0.526	1.018	0.416	0.881	0.511

Note: * Significant at the *p* < 0.05 level; ** Significant at the *p* < 0.01 level; *** Significant at the *p* < 0.001 level.

**Table 4:** Understorey characteristics, litter thickness, soil temperature, and moisture content (mean ± SD) across different stand types.

Characteristics	Broadleaf	Coniferous	Mixed	Secondary	F	*p*-Value
Plant richness (species·m^−2^)	6.00 ± 1.06	3.80 ± 0.92	4.35 ± 0.48	5.70 ± 0.76	1.679	*p* = 0.183
Plant coverage (%)	56.70 ± 12.52	32.30 ± 9.17	45.35 ± 6.97	45.80 ± 10.56	1.054	*p* = 0.377
Shannon–Wiener index (understory)	1.03 ± 0.21	0.65 ± 0.17	0.92 ± 0.11	0.92 ± 0.08	2.182	*p* = 0.102
Simpson index (understory)	0.48 ± 0.09 ab	0.32 ± 0.08 b	0.41 ± 0.06 ab	0.61 ± 0.04 a	2.769	*p* = 0.050 *
LT/cm	2.50 ± 0.20 b	4.05 ± 0.27 a	3.13 ± 0.17 b	2.70 ± 0.22 b	3.258	*p* < 0.029 **
Tem /℃	18.18 ± 0.14 a	16.92 ± 0.20 b	18.22 ± 0.08 a	17.89 ± 0.07	22.528	*p* < 0.000 ***
Mois (%)	30.25 ± 1.53 a	25.28 ± 0.68 b	24.57 ± 1.18 b	27.89 ± 1.20 b	7.606	*p* < 0.000 ***

Note: Lowercase letters indicate significant differences between stands. *, **, and *** indicate significant differences at the *p* < 0.05 level, *p* < 0.01 level, and *p* < 0.001 level, respectively. Abbreviations: LT: litter thickness, Tem: Soil temperature, Mois: Soil moisture.

**Table 5:** Soil chemical properties (mean ± SD) across different stand types.

Characteristics	Broadleaf	Coniferous	Mixed	Secondary	F	*p*-Value
pH (H_2_O)	5.50 ± 0.08 b	5.58 ± 0.03 b	5.68 ± 0.06 b	5.92 ± 0.06 a	7.241	*p* < 0.00 ***
TC (mg·g^–1^)	8.06 ± 0.43	7.88 ± 0.33	9.34 ± 0.66	7.47 ± 0.77	2.067	*p* = 0.116
TN (mg·g^–1^)	1.29 ± 0.21 a	0.72 ± 0.03 b	0.92 ± 0.15 ab	0.67 ± 0.06 b	3.161	*p* = 0.032 *
C/N ratio	7.64 ± 1.05 b	10.80 ± 0.16 a	10.23 ± 0.49 a	11.33 ± 0.16 a	7.196	*p* < 0.000 ***
TP (mg·g^–1^)	3.92 ± 0.35 b	2.97 ± 0.11 b	3.53 ± 0.17 b	5.45 ± 0.75 a	8.732	*p* < 0.00 ***
TK (mg·g^–1^)	41.32 ± 2.68	37.40 ± 1.83	37.65 ± 1.32	36.68 ± 1.18	1.075	*p* = 0.367
AP (mg·g^–1^)	30.72 ± 3.31 b	34.60 ± 2.48 b	39.27 ± 2.21 b	80.28 ± 5.01 a	44.372	*p* < 0.00 ***
AK (mg·g^–1^)	230.77 ± 19.14 b	216.64 ± 12.47 b	219.48 ± 12.17 b	279.22 ± 17.76 a	3.277	*p* = 0.028 *
NO_3_^−^ (mg·kg^–1^)	5.66 ± 0.63 b	11.58 ± 1.13 a	14.13 ± 0.83 a	14.01 ± 1.36 a	12.425	*p* < 0.00 ***
NH_4_^+^ (mg·kg^–1^)	22.35 ± 1.36 a	18.81 ± 0.59 b	22.24 ± 0.89 a	20.54 ± 1.21 a	3.100	*p* = 0.035 *

Note: Lowercase letters indicate significant differences between stands. * and *** indicate significant differences at the *p* < 0.05 level, and *p* < 0.001 level, respectively. Abbreviations: pH: Soil pH, TC: Total carbon, TN: Total nitrogen, TP: Total phosphorus, TK: Total potassium, AP: available phosphorus, AK: quick-acting potassium, NO_3_^−^: nitrate nitrogen, NH_4_^+^: Ammonium nitrogen.

## Data Availability

The data are contained within the article or the Supplementary Tables.

## Supplementary Materials

The following supporting information can be downloaded at: https://www.mdpi.com/article/10.3390/biology15141198/s1, Table S1. List of soil meso- and micro-fauna and the number of individual identified in the different seasons and stand types. Table S2. Understory characteristics, litter thickness, soil temperature and moisture content (mean ± SD) of 11 stands. Table S3. Soil chemical properties (mean ± SD) of the 11 stands. Table S4. Results of redundancy analysis model of soil meso- and micro-fauna community variation using biological and abiotic environmental variables, determined by forward selection procedure with unrestricted permutation tests.
