# Supplementary material for: Mixed Plantations Mitigate Negative Effects of Natural Forest Conversion on Soil Meso- and Micro-Faunal Communities in Zhangguangcailing Mountains, Northeast China
Source: Biology (Basel). 2026 Jul 20;15(14):1198. doi: 10.3390/biology15141198 (PMC13404701; doi:10.3390/biology15141198)
Supplement: Supplementary file 1 [file biology-15-01198-s001.zip › biology-4429000-supplementary.pdf]

Supplementary Materials Table S1–S4.

Article title: Mixed plantations mitigate negative effects of natural forest conversion on soil meso- and micro-faunal communities in Zhangguangcailing mountains, Northeast China

Authors: Shuangjiao Ma[1,#], Yong Zhang[2,#], Kun Li[1], Qingcheng Wang[3], Donghai Cui[3], Zengwang Yao[1], Chuanrong Li[1,*] and Yehan Tian[1,*]

[1]Mountain Tai Forest Ecosystem Research Station of State Forestry Administration, Research Center for Forest Carbon Neutrality Engineering of Shandong Higher Education Institutions, Tai'an271018, China.
[2]College of Forestry, Southwest Forestry University, Kunming 650224, China
[3]College of Forestry, Northeast Forestry University, Harbin150040, China

* **Corresponding author:** Chuanrong Li, E-mail: chrli@sdau.edu.cn; Yehan Tian, Email: tianyehan@163.com.

Table S1. List of soil meso- and micro-fauna and the number of individual identified in the different seasons and stand types.

Total		FM		JM		LG		PS		PA		FM-LG		FM-PS		JM-LG		JM-PS		SF-1		SF-2		Total	
Taxonomic group	Trophic group	Ind	P (%)	Ind	P (%)	Ind	P (%)	Ind	P (%)	Ind	P (%)	Ind	P (%)	Ind	P (%)	Ind	P (%)	Ind	P (%)	Ind	P (%)	Ind	P (%)	Ind	P (%)
Oribatida	S	8903	63.00	12841	63.75	3944	44.27	5282	45.34	5820	41.82	6726	50.52	7097	47.48	10406	59.86	9320	50.09	8564	51.11	7423	50.88	86326	52.53
Onychiuridae	S	1066	7.54	1391	6.91	1423	15.97	1851	15.89	1269	9.12	1684	12.65	1554	10.40	1250	7.19	1714	9.21	3000	17.90	1620	11.10	17822	10.84
Isotomidae	S	979	6.93	1639	8.14	905	10.16	1594	13.68	1858	13.35	674	5.06	2057	13.76	1339	7.70	2227	11.97	1423	8.49	1055	7.23	15750	9.58
Mesostigmata	Pr	866	6.13	1297	6.44	1176	13.20	1150	9.87	1815	13.04	1088	8.17	1394	9.33	1246	7.17	1413	7.59	1134	6.77	1247	8.55	13826	8.41
Diptera larvae	S	1148	8.12	801	3.98	493	5.53	549	4.71	767	5.51	608	4.57	721	4.82	1053	6.06	996	5.35	790	4.71	1309	8.97	9235	5.62
Prostigmata	Pr	297	2.10	714	3.54	231	2.59	241	2.07	473	3.40	873	6.56	863	5.77	367	2.11	813	4.37	643	3.84	508	3.48	6023	3.66
Hypogastruridae	S	368	2.60	553	2.75	141	1.58	286	2.46	636	4.57	1062	7.98	447	2.99	598	3.44	773	4.15	553	3.30	517	3.54	5934	3.61
Enchytraeidae	S	191	1.35	269	1.34	141	1.58	155	1.33	195	1.40	180	1.35	193	1.29	284	1.63	308	1.66	215	1.28	234	1.60	2365	1.44
Neanridae	Pr	73	0.52	128	0.64	66	0.74	112	0.96	286	2.06	56	0.42	91	0.61	127	0.73	167	0.90	64	0.38	105	0.72	1275	0.78
Tomoceridae	S	20	0.14	170	0.84	41	0.46	51	0.44	83	0.60	46	0.35	77	0.52	119	0.68	187	1.00	67	0.40	30	0.21	891	0.54
Entomobryidae	S	14	0.10	30	0.15	33	0.37	111	0.95	43	0.31	43	0.32	50	0.33	39	0.22	129	0.69	26	0.16	11	0.08	529	0.32
Protura	F	7	0.05	7	0.03	43	0.48	35	0.30	92	0.66	30	0.23	119	0.80	33	0.19	52	0.28	5	0.03	65	0.45	488	0.30
Lithobiomorpha	Pr	13	0.09	30	0.15	13	0.15	33	0.28	34	0.24	16	0.12	59	0.39	36	0.21	60	0.32	20	0.12	99	0.68	413	0.25
Formicidae	O	12	0.08	19	0.09	28	0.31	26	0.22	2	0.01	60	0.45	11	0.07	129	0.74	17	0.09	13	0.08	61	0.42	378	0.23
Stylommatophora	S	19	0.13	31	0.15	7	0.08	31	0.27	63	0.45	8	0.06	22	0.15	42	0.24	77	0.41	44	0.26	20	0.14	364	0.22
Sminthuridae	S	16	0.11	31	0.15	9	0.10	11	0.09	11	0.08	1	0.01	25	0.17	56	0.32	126	0.68	23	0.14	6	0.04	315	0.19
Hirudisomatidae	S	2	0.01	40	0.20	27	0.30	20	0.17	9	0.06	19	0.14	27	0.18	25	0.14	25	0.13	15	0.09	35	0.24	244	0.15

Hemiptera larvae	Ph	11	0.08	1	0.00	27	0.30	0	0.00	179	1.29	2	0.02	5	0.03	4	0.02	4	0.02	1	0.01	1	0.01	235	0.14
Scutigerellidae	S	7	0.05	12	0.06	17	0.19	24	0.21	25	0.18	21	0.16	35	0.23	9	0.05	18	0.10	12	0.07	25	0.17	205	0.12
Staphylinidae	S	17	0.12	21	0.10	10	0.11	10	0.09	19	0.14	20	0.15	12	0.08	12	0.07	15	0.08	34	0.20	4	0.03	174	0.11
Geophilomorpha	Pr	5	0.04	11	0.05	17	0.19	6	0.05	19	0.14	17	0.13	13	0.09	30	0.17	10	0.05	7	0.04	19	0.13	154	0.09
Coleoptera larvae	Pr	7	0.05	6	0.03	0	0.00	0	0.00	94	0.68	4	0.03	3	0.02	8	0.05	19	0.10	4	0.02	1	0.01	146	0.09
Curculionidae larvae	Ph	0	0.00	1	0.00	0	0.00	13	0.11	9	0.06	27	0.20	2	0.01	23	0.13	2	0.01	4	0.02	65	0.45	146	0.09
Ptiliidae	S	14	0.10	3	0.01	22	0.25	13	0.11	15	0.11	9	0.07	5	0.03	13	0.07	16	0.09	7	0.04	17	0.12	134	0.08
Chthonidae	Pr	2	0.01	17	0.08	1	0.01	3	0.03	6	0.04	0	0.00	11	0.07	30	0.17	19	0.10	5	0.03	29	0.20	123	0.07
Staphylinidae larvae	S	23	0.16	6	0.03	3	0.03	3	0.03	17	0.12	4	0.03	3	0.02	5	0.03	8	0.04	5	0.03	11	0.08	88	0.05
Neelidae	S	0	0.00	0	0.00	6	0.07	9	0.08	18	0.13	1	0.01	7	0.05	11	0.06	16	0.09	9	0.05	6	0.04	83	0.05
Elatcridae larvae	Ph	4	0.03	6	0.03	9	0.10	2	0.02	7	0.05	3	0.02	12	0.08	15	0.09	11	0.06	2	0.01	7	0.05	78	0.05
Araneidae	Pr	4	0.03	13	0.06	10	0.11	7	0.06	9	0.06	0	0.00	2	0.01	15	0.09	4	0.02	3	0.02	5	0.03	72	0.04
Pselaphidae	Pr	9	0.06	3	0.01	1	0.01	0	0.00	2	0.01	4	0.03	0	0.00	10	0.06	8	0.04	16	0.10	0	0.00	53	0.03
Cicadelloidea	Ph	4	0.03	8	0.04	11	0.12	0	0.00	6	0.04	2	0.02	2	0.01	1	0.01	3	0.02	7	0.04	1	0.01	45	0.03
Pauropoda	F	2	0.01	0	0.00	6	0.07	4	0.03	9	0.06	5	0.04	1	0.01	5	0.03	0	0.00	2	0.01	10	0.07	44	0.03
Lepidoptera larvae	Ph	1	0.01	2	0.01	2	0.02	1	0.01	0	0.00	2	0.02	2	0.01	6	0.03	7	0.04	5	0.03	1	0.01	29	0.02
Thysanoptera	F	0	0.00	5	0.02	0	0.00	0	0.00	0	0.00	3	0.02	1	0.01	3	0.02	2	0.01	7	0.04	2	0.01	23	0.01
Carabidae	Pr	3	0.02	3	0.01	2	0.02	1	0.01	1	0.01	1	0.01	1	0.01	1	0.01	2	0.01	0	0.00	7	0.05	22	0.01
Lumbricidae	S	0	0.00	2	0.01	8	0.09	5	0.04	5	0.04	1	0.01	0	0.00	0	0.00	0	0.00	0	0.00	0	0.00	21	0.01
Scaphidiidae	S	1	0.01	1	0.00	3	0.03	1	0.01	1	0.01	1	0.01	1	0.01	5	0.03	0	0.00	4	0.02	2	0.01	20	0.01
Lampyridae larvae	Pr	2	0.01	1	0.00	0	0.00	0	0.00	2	0.01	2	0.02	3	0.02	2	0.01	6	0.03	2	0.01	0	0.00	20	0.01
Silphidae	S	2	0.01	1	0.00	2	0.02	1	0.01	2	0.01	1	0.01	0	0.00	3	0.02	1	0.01	4	0.02	1	0.01	18	0.01
Carabidae larvae	Pr	6	0.04	1	0.00	4	0.04	1	0.01	0	0.00	0	0.00	0	0.00	1	0.01	0	0.00	0	0.00	3	0.02	16	0.01
Chrysomelidae	Ph	0	0.00	1	0.00	4	0.04	1	0.01	2	0.01	0	0.00	1	0.01	0	0.00	2	0.01	2	0.01	1	0.01	14	0.01

larvae																									
Nitidulidae larvae	Pr	0	0.00	1	0.00	2	0.02	0	0.00	0	0.00	0	0.00	1	0.01	3	0.02	3	0.02	0	0.00	4	0.03	14	0.01
Cantharidae larvae	Pr	0	0.00	1	0.00	0	0.00	1	0.01	2	0.01	5	0.04	1	0.01	1	0.01	1	0.01	1	0.01	0	0.00	13	0.01
Lycosidae	Pr	1	0.01	6	0.03	0	0.00	0	0.00	0	0.00	0	0.00	0	0.00	1	0.01	2	0.01	3	0.02	0	0.00	13	0.01
Mycetophagidae	S	0	0.00	1	0.00	1	0.01	0	0.00	2	0.01	0	0.00	0	0.00	0	0.00	5	0.03	2	0.01	0	0.00	11	0.01
Hahniidae	Pr	0	0.00	3	0.01	0	0.00	0	0.00	0	0.00	0	0.00	0	0.00	2	0.01	3	0.02	0	0.00	1	0.01	9	0.01
Curculionidae	Ph	2	0.01	0	0.00	2	0.02	0	0.00	0	0.00	1	0.01	2	0.01	0	0.00	1	0.01	0	0.00	0	0.00	8	0.00
Scarabaeidae	S	1	0.01	1	0.00	0	0.00	0	0.00	1	0.01	0	0.00	0	0.00	0	0.00	1	0.01	1	0.01	3	0.02	8	0.00
Chrysomelidae	Ph	0	0.00	0	0.00	1	0.01	0	0.00	1	0.01	0	0.00	2	0.01	1	0.01	2	0.01	0	0.00	0	0.00	7	0.00
Anthocoridae	Ph	0	0.00	3	0.01	1	0.01	0	0.00	0	0.00	0	0.00	0	0.00	0	0.00	0	0.00	2	0.01	1	0.01	7	0.00
Paradoxosomatidae	S	2	0.01	0	0.00	0	0.00	0	0.00	0	0.00	0	0.00	1	0.01	2	0.01	1	0.01	0	0.00	1	0.01	7	0.00
Nitidulidae	S	0	0.00	0	0.00	0	0.00	0	0.00	2	0.01	1	0.01	1	0.01	0	0.00	0	0.00	1	0.01	2	0.01	7	0.00
Hymenoptera	Ph	0	0.00	0	0.00	0	0.00	0	0.00	0	0.00	0	0.00	2	0.01	0	0.00	1	0.01	1	0.01	3	0.02	7	0.00
Lepidoptera larvae	Ph	1	0.01	0	0.00	3	0.03	0	0.00	0	0.00	0	0.00	0	0.00	0	0.00	1	0.01	0	0.00	1	0.01	6	0.00
Scolytidae	Ph	4	0.03	0	0.00	1	0.01	0	0.00	0	0.00	0	0.00	0	0.00	0	0.00	1	0.01	0	0.00	0	0.00	6	0.00
Psocoptera	Ph	0	0.00	2	0.01	0	0.00	0	0.00	1	0.01	0	0.00	1	0.01	0	0.00	2	0.01	0	0.00	0	0.00	6	0.00
Scydmaenidae	Pr	0	0.00	2	0.01	0	0.00	0	0.00	1	0.01	0	0.00	1	0.01	0	0.00	1	0.01	0	0.00	1	0.01	6	0.00
Diplura	Pr	0	0.00	0	0.00	0	0.00	2	0.02	0	0.00	0	0.00	0	0.00	1	0.01	0	0.00	0	0.00	3	0.02	6	0.00
Microphysidae	Ph	0	0.00	0	0.00	0	0.00	0	0.00	0	0.00	0	0.00	1	0.01	1	0.01	0	0.00	1	0.01	3	0.02	6	0.00
Hebridae	Ph	0	0.00	0	0.00	3	0.03	0	0.00	0	0.00	0	0.00	1	0.01	0	0.00	1	0.01	0	0.00	0	0.00	5	0.00
Tenebrionidae	Pr	0	0.00	0	0.00	0	0.00	0	0.00	3	0.02	0	0.00	2	0.01	0	0.00	0	0.00	0	0.00	0	0.00	5	0.00
Clubionidae	Pr	0	0.00	2	0.01	1	0.01	0	0.00	0	0.00	0	0.00	0	0.00	0	0.00	0	0.00	1	0.01	1	0.01	5	0.00
Thomisidae	Pr	0	0.00	0	0.00	0	0.00	0	0.00	0	0.00	0	0.00	0	0.00	2	0.01	2	0.01	0	0.00	0	0.00	4	0.00
Coccinellidae	Pr	0	0.00	0	0.00	1	0.01	0	0.00	0	0.00	0	0.00	2	0.01	0	0.00	0	0.00	0	0.00	0	0.00	3	0.00
Pselaphidae larvae	Pr	1	0.01	0	0.00	0	0.00	1	0.01	0	0.00	1	0.01	0	0.00	0	0.00	0	0.00	0	0.00	0	0.00	3	0.00
Zodariidae	Pr	0	0.00	0	0.00	0	0.00	1	0.01	0	0.00	0	0.00	0	0.00	2	0.01	0	0.00	0	0.00	0	0.00	3	0.00

Saltieidae	Pr	0	0.00	0	0.00	0	0.00	0	0.00	0	0.00	0	0.00	0	0.00	1	0.01	1	0.01	0	0.00	1	0.01	3	0.00
Psgllipsocidae	Pr	0	0.00	0	0.00	2	0.02	0	0.00	0	0.00	0	0.00	0	0.00	0	0.00	0	0.00	0	0.00	0	0.00	2	0.00
Silphidae	S	0	0.00	0	0.00	2	0.02	0	0.00	0	0.00	0	0.00	0	0.00	0	0.00	0	0.00	0	0.00	0	0.00	2	0.00
Liocranidae	Pr	1	0.01	0	0.00	1	0.01	0	0.00	0	0.00	0	0.00	0	0.00	0	0.00	0	0.00	0	0.00	0	0.00	2	0.00
Cydnidae	Ph	0	0.00	1	0.00	1	0.01	0	0.00	0	0.00	0	0.00	0	0.00	0	0.00	0	0.00	0	0.00	0	0.00	2	0.00
Chelonariidae	S	0	0.00	0	0.00	0	0.00	0	0.00	0	0.00	0	0.00	0	0.00	2	0.01	0	0.00	0	0.00	0	0.00	2	0.00
Cantharidae	Pr	0	0.00	0	0.00	0	0.00	0	0.00	0	0.00	0	0.00	0	0.00	1	0.01	1	0.01	0	0.00	0	0.00	2	0.00
Pyrrhocoridae	Ph	0	0.00	1	0.00	0	0.00	0	0.00	0	0.00	0	0.00	0	0.00	0	0.00	0	0.00	1	0.01	0	0.00	2	0.00
Lygaeidae	Ph	0	0.00	0	0.00	0	0.00	0	0.00	0	0.00	0	0.00	0	0.00	0	0.00	0	0.00	1	0.01	1	0.01	2	0.00
Lampyridae	Pr	0	0.00	0	0.00	0	0.00	1	0.01	0	0.00	0	0.00	0	0.00	0	0.00	0	0.00	0	0.00	0	0.00	1	0.00
Erotylidae	S	0	0.00	0	0.00	0	0.00	0	0.00	0	0.00	1	0.01	0	0.00	0	0.00	0	0.00	0	0.00	0	0.00	1	0.00
Elatcridae	Ph	0	0.00	0	0.00	0	0.00	0	0.00	0	0.00	0	0.00	1	0.01	0	0.00	0	0.00	0	0.00	0	0.00	1	0.00
Deramptera	O	0	0.00	0	0.00	0	0.00	0	0.00	0	0.00	0	0.00	0	0.00	0	0.00	0	0.00	1	0.01	0	0.00	1	0.00
Zoridae	Pr	0	0.00	0	0.00	0	0.00	0	0.00	0	0.00	0	0.00	0	0.00	1	0.01	0	0.00	0	0.00	0	0.00	1	0.00
Hydrophilidae	S	0	0.00	0	0.00	0	0.00	0	0.00	0	0.00	0	0.00	0	0.00	1	0.01	0	0.00	0	0.00	0	0.00	1	0.00
Endomychidae	S	0	0.00	0	0.00	0	0.00	0	0.00	0	0.00	0	0.00	0	0.00	1	0.01	0	0.00	0	0.00	0	0.00	1	0.00
Silvanidae	Ph	0	0.00	0	0.00	0	0.00	0	0.00	0	0.00	0	0.00	0	0.00	0	0.00	1	0.01	0	0.00	0	0.00	1	0.00
Cleridae larvae	Pr	0	0.00	1	0.00	0	0.00	0	0.00	0	0.00	0	0.00	0	0.00	0	0.00	0	0.00	0	0.00	0	0.00	1	0.00
Agelenidae	Pr	0	0.00	1	0.00	0	0.00	0	0.00	0	0.00	0	0.00	0	0.00	0	0.00	0	0.00	0	0.00	0	0.00	1	0.00
Gnaphosidae	Pr	0	0.00	0	0.00	0	0.00	0	0.00	0	0.00	0	0.00	0	0.00	0	0.00	0	0.00	0	0.00	1	0.01	1	0.00
Taxa number		44	/	53	/	51	/	40	/	46	/	42	/	51	/	55	/	56	/	50	/	53	/	86	/
Total individual number		14131	/	20142	/	8908	/	11649	/	13916	/	13313	/	14948	/	17384	/	18608	/	16757	/	14590	/	164346	/

June		FM	JM	LG	PS	PA	FM-LG	FM-PS	JM-LG	JM-PS	SF-1	SF-2	Total

Taxonomic group	Trophic group	Ind	P (%)	Ind	P (%)	Ind	P (%)	Ind	P (%)	Ind	P (%)	Ind	P (%)	Ind	P (%)	Ind	P (%)	Ind	P (%)	Ind	P (%)	Ind	P (%)	Ind	P (%)
Oribatida	S	1525	57.94	5979	74.50	1183	53.80	682	37.76	2256	52.32	1573	54.64	2747	55.72	4026	69.65	3247	55.86	2907	63.71	2179	50.73	28304	59.92
Onychiuridae	S	198	7.52	428	5.33	233	10.60	474	26.25	209	4.85	304	10.56	432	8.76	383	6.63	507	8.72	452	9.91	477	11.11	4097	8.67
Mesostigmata	Pr	143	5.43	354	4.41	219	9.96	171	9.47	547	12.69	232	8.06	329	6.67	323	5.59	300	5.16	220	4.82	278	6.47	3116	6.60
Diptera larvae	S	201	7.64	238	2.97	193	8.78	181	10.02	283	6.56	111	3.86	234	4.75	267	4.62	376	6.47	265	5.81	291	6.78	2640	5.59
Isotomidae	S	294	11.17	164	2.04	151	6.87	74	4.10	310	7.19	99	3.44	340	6.90	243	4.20	513	8.83	145	3.18	199	4.63	2532	5.36
Prostigmata	Pr	104	3.95	418	5.21	92	4.18	82	4.54	138	3.20	170	5.90	492	9.98	190	3.29	288	4.95	237	5.19	310	7.22	2521	5.34
Hypogastruridae	S	74	2.81	193	2.40	21	0.95	31	1.72	96	2.23	151	5.24	122	2.47	51	0.88	157	2.70	202	4.43	152	3.54	1250	2.65
Enchytraeidae	S	30	1.14	122	1.52	37	1.68	44	2.44	103	2.39	83	2.88	96	1.95	67	1.16	150	2.58	71	1.56	126	2.93	929	1.97
Formicidae	O	2	0.08	8	0.10	1	0.05	16	0.89	1	0.02	55	1.91	6	0.12	89	1.54	10	0.17	3	0.07	57	1.33	248	0.53
Neanridae	Pr	5	0.19	30	0.37	3	0.14	7	0.39	44	1.02	10	0.35	29	0.59	21	0.36	30	0.52	16	0.35	21	0.49	216	0.46
Protura	F	2	0.08	3	0.04	9	0.41	5	0.28	51	1.18	17	0.59	29	0.59	7	0.12	21	0.36	0	0.00	12	0.28	156	0.33
Lithobiomorpha	Pr	7	0.27	8	0.10	0	0.00	0	0.00	16	0.37	4	0.14	21	0.43	11	0.19	26	0.45	3	0.07	29	0.68	125	0.26
Coleoptera larvae	Pr	5	0.19	0	0.00	0	0.00	0	0.00	94	2.18	2	0.07	3	0.06	3	0.05	16	0.28	1	0.02	1	0.02	125	0.26
Curculionidae larvae	Ph	0	0.00	0	0.00	0	0.00	11	0.61	0	0.00	27	0.94	0	0.00	18	0.31	0	0.00	0	0.00	63	1.47	119	0.25
Tomoceridae	S	0	0.00	13	0.16	0	0.00	1	0.06	44	1.02	0	0.00	1	0.02	5	0.09	34	0.58	1	0.02	0	0.00	99	0.21
Sminthuridae	S	8	0.30	5	0.06	7	0.32	3	0.17	9	0.21	0	0.00	1	0.02	5	0.09	33	0.57	2	0.04	5	0.12	78	0.17
Geophilomorpha	Pr	0	0.00	7	0.09	6	0.27	1	0.06	10	0.23	3	0.10	8	0.16	14	0.24	6	0.10	0	0.00	9	0.21	64	0.14
Staphylinidae	S	6	0.23	0	0.00	2	0.09	1	0.06	11	0.26	14	0.49	6	0.12	6	0.10	5	0.09	8	0.18	2	0.05	61	0.13

Stylommatophora	S	0	0.00	9	0.11	0	0.00	0	0.00	16	0.37	1	0.03	2	0.04	5	0.09	11	0.19	6	0.13	10	0.23	60	0.13
Staphylinidae larvae	S	14	0.53	4	0.05	2	0.09	2	0.11	9	0.21	1	0.03	1	0.02	3	0.05	5	0.09	1	0.02	10	0.23	52	0.11
Neelidae	S	0	0.00	0	0.00	2	0.09	3	0.17	12	0.28	0	0.00	3	0.06	4	0.07	13	0.22	2	0.04	6	0.14	45	0.10
Entomobryidae	S	0	0.00	2	0.02	2	0.09	2	0.11	11	0.26	2	0.07	2	0.04	0	0.00	20	0.34	0	0.00	4	0.09	45	0.10
Hirudisomatidae	S	0	0.00	3	0.04	3	0.14	0	0.00	0	0.00	1	0.03	5	0.10	9	0.16	6	0.10	1	0.02	11	0.26	39	0.08
Scutigerellidae	S	1	0.04	2	0.02	5	0.23	5	0.28	8	0.19	3	0.10	1	0.02	2	0.03	1	0.02	2	0.04	6	0.14	36	0.08
Ptiliidae	S	2	0.08	1	0.01	9	0.41	0	0.00	1	0.02	3	0.10	1	0.02	2	0.03	11	0.19	5	0.11	1	0.02	36	0.08
Pauropoda	F	0	0.00	0	0.00	2	0.09	3	0.17	7	0.16	5	0.17	1	0.02	2	0.03	0	0.00	0	0.00	7	0.16	27	0.06
Araneidae	Pr	2	0.08	10	0.12	1	0.05	0	0.00	0	0.00	0	0.00	0	0.00	7	0.12	2	0.03	0	0.00	2	0.05	24	0.05
Chthonidae	Pr	0	0.00	1	0.01	0	0.00	0	0.00	0	0.00	0	0.00	1	0.02	8	0.14	3	0.05	0	0.00	10	0.23	23	0.05
Hemiptera larvae	Ph	5	0.19	0	0.00	3	0.14	0	0.00	9	0.21	0	0.00	0	0.00	1	0.02	1	0.02	0	0.00	1	0.02	20	0.04
Elatcridae larvae	Ph	0	0.00	3	0.04	2	0.09	1	0.06	1	0.02	2	0.07	2	0.04	1	0.02	0	0.00	0	0.00	2	0.05	14	0.03
Cicadelloidea	Ph	0	0.00	5	0.06	1	0.05	0	0.00	1	0.02	2	0.07	2	0.04	0	0.00	1	0.02	2	0.04	0	0.00	14	0.03
Carabidae	Pr	2	0.08	1	0.01	0	0.00	1	0.06	1	0.02	0	0.00	0	0.00	1	0.02	1	0.02	0	0.00	3	0.07	10	0.02
Lumbricidae	S	0	0.00	1	0.01	1	0.05	2	0.11	4	0.09	0	0.00	0	0.00	0	0.00	0	0.00	0	0.00	0	0.00	8	0.02
Thysanoptera	F	0	0.00	4	0.05	0	0.00	0	0.00	0	0.00	0	0.00	0	0.00	0	0.00	1	0.02	1	0.02	1	0.02	7	0.01
Hymenoptera	Ph	0	0.00	0	0.00	0	0.00	0	0.00	0	0.00	0	0.00	2	0.04	0	0.00	1	0.02	1	0.02	3	0.07	7	0.01
Lampyridae larvae	Pr	1	0.04	0	0.00	0	0.00	0	0.00	2	0.05	0	0.00	1	0.02	0	0.00	3	0.05	0	0.00	0	0.00	7	0.01
Silphidae	S	0	0.00	0	0.00	0	0.00	0	0.00	2	0.05	1	0.03	0	0.00	2	0.03	1	0.02	1	0.02	0	0.00	7	0.01
Anthocoridae	Ph	0	0.00	3	0.04	0	0.00	0	0.00	0	0.00	0	0.00	0	0.00	0	0.00	0	0.00	2	0.04	1	0.02	6	0.01
Microphysidae	Ph	0	0.00	0	0.00	0	0.00	0	0.00	0	0.00	0	0.00	1	0.02	1	0.02	0	0.00	1	0.02	3	0.07	6	0.01
Mycetophagidae	S	0	0.00	0	0.00	0	0.00	0	0.00	1	0.02	0	0.00	0	0.00	0	0.00	5	0.09	0	0.00	0	0.00	6	0.01
Cantharidae larvae	Pr	0	0.00	1	0.01	0	0.00	1	0.06	1	0.02	1	0.03	0	0.00	0	0.00	0	0.00	1	0.02	0	0.00	5	0.01
Diplura	Pr	0	0.00	0	0.00	0	0.00	2	0.11	0	0.00	0	0.00	0	0.00	0	0.00	0	0.00	0	0.00	3	0.07	5	0.01
Pselaphidae	Pr	0	0.00	0	0.00	1	0.05	0	0.00	1	0.02	0	0.00	0	0.00	0	0.00	3	0.05	0	0.00	0	0.00	5	0.01

Lepidoptera larvae	Ph	1	0.04	0	0.00	2	0.09	0	0.00	0	0.00	0	0.00	0	0.00	0	0.00	1	0.02	0	0.00	0	0.00	4	0.01
Nitidulidae larvae	Pr	0	0.00	1	0.01	0	0.00	0	0.00	0	0.00	0	0.00	1	0.02	1	0.02	1	0.02	0	0.00	0	0.00	4	0.01
Chrysomelidae larvae	Ph	0	0.00	0	0.00	4	0.18	0	0.00	0	0.00	0	0.00	0	0.00	0	0.00	0	0.00	0	0.00	0	0.00	4	0.01
Psocoptera	Ph	0	0.00	1	0.01	0	0.00	0	0.00	1	0.02	0	0.00	0	0.00	0	0.00	1	0.02	0	0.00	0	0.00	3	0.01
Nitidulidae	S	0	0.00	0	0.00	0	0.00	0	0.00	1	0.02	0	0.00	1	0.02	0	0.00	0	0.00	0	0.00	0	0.00	2	0.00
Tenebrionidae	Pr	0	0.00	0	0.00	0	0.00	0	0.00	0	0.00	0	0.00	2	0.04	0	0.00	0	0.00	0	0.00	0	0.00	2	0.00
Coccinellidae	Pr	0	0.00	0	0.00	0	0.00	0	0.00	0	0.00	0	0.00	2	0.04	0	0.00	0	0.00	0	0.00	0	0.00	2	0.00
Paradoxosomatidae	S	0	0.00	0	0.00	0	0.00	0	0.00	0	0.00	0	0.00	1	0.02	1	0.02	0	0.00	0	0.00	0	0.00	2	0.00
Psgllipsocidae	Ph	0	0.00	0	0.00	2	0.09	0	0.00	0	0.00	0	0.00	0	0.00	0	0.00	0	0.00	0	0.00	0	0.00	2	0.00
Curculionidae	Ph	0	0.00	0	0.00	0	0.00	0	0.00	0	0.00	1	0.03	1	0.02	0	0.00	0	0.00	0	0.00	0	0.00	2	0.00
Scaphidiidae	S	0	0.00	0	0.00	0	0.00	0	0.00	0	0.00	0	0.00	0	0.00	1	0.02	0	0.00	0	0.00	0	0.00	1	0.00
Erotylidae	S	0	0.00	0	0.00	0	0.00	0	0.00	0	0.00	1	0.03	0	0.00	0	0.00	0	0.00	0	0.00	0	0.00	1	0.00
Deramptera	O	0	0.00	0	0.00	0	0.00	0	0.00	0	0.00	0	0.00	0	0.00	0	0.00	0	0.00	1	0.02	0	0.00	1	0.00
Pyrrhocoridae	Ph	0	0.00	0	0.00	0	0.00	0	0.00	0	0.00	0	0.00	0	0.00	0	0.00	0	0.00	1	0.02	0	0.00	1	0.00
Cantharidae	Pr	0	0.00	0	0.00	0	0.00	0	0.00	0	0.00	0	0.00	0	0.00	0	0.00	1	0.02	0	0.00	0	0.00	1	0.00
Scarabaeidae	S	0	0.00	0	0.00	0	0.00	0	0.00	0	0.00	0	0.00	0	0.00	0	0.00	0	0.00	1	0.02	0	0.00	1	0.00
Lycosidae	Pr	0	0.00	1	0.01	0	0.00	0	0.00	0	0.00	0	0.00	0	0.00	0	0.00	0	0.00	0	0.00	0	0.00	1	0.00
Lepidoptera larvae	Ph	0	0.00	0	0.00	0	0.00	0	0.00	0	0.00	0	0.00	0	0.00	0	0.00	1	0.02	0	0.00	0	0.00	1	0.00
Hebridae	Ph	0	0.00	0	0.00	0	0.00	0	0.00	0	0.00	0	0.00	1	0.02	0	0.00	0	0.00	0	0.00	0	0.00	1	0.00
Scydmaenidae	Pr	0	0.00	1	0.01	0	0.00	0	0.00	0	0.00	0	0.00	0	0.00	0	0.00	0	0.00	0	0.00	0	0.00	1	0.00
Cydnidae	Ph	0	0.00	1	0.01	0	0.00	0	0.00	0	0.00	0	0.00	0	0.00	0	0.00	0	0.00	0	0.00	0	0.00	1	0.00
Chrysomelidae	Ph	0	0.00	0	0.00	0	0.00	0	0.00	1	0.02	0	0.00	0	0.00	0	0.00	0	0.00	0	0.00	0	0.00	1	0.00
Hahniidae	Pr	0	0.00	1	0.01	0	0.00	0	0.00	0	0.00	0	0.00	0	0.00	0	0.00	0	0.00	0	0.00	0	0.00	1	0.00
Lygaeidae	Ph	0	0.00	0	0.00	0	0.00	0	0.00	0	0.00	0	0.00	0	0.00	0	0.00	0	0.00	1	0.02	0	0.00	1	0.00
Taxa number		23	/	36	/	30	/	26	/	37	/	29	/	38	/	35	/	40	/	32	/	35	/	67	/

Total individual number		2632	/	8026	/	2199	/	1806	/	4312	/	2879	/	4930	/	5780	/	5813	/	4563	/	4295	/	47235	/

August		FM		JM		LG		PS		PA		FM-LG		FM-PS		JM-LG		JM-PS		SF-1		SF-2		Total	
Taxonomic group	Trophic group	Ind	P (%)	Ind	P (%)	Ind	P (%)	Ind	P (%)	Ind	P (%)	Ind	P (%)	Ind	P (%)	Ind	P (%)	Ind	P (%)	Ind	P (%)	Ind	P (%)	Ind	P (%)
Oribatida	S	5323	65.72	3388	54.81	1221	43.79	2298	43.51	1840	39.80	2819	48.35	2378	38.65	2647	51.96	3049	43.08	2960	43.82	2640	49.09	30563	48.31
Isotomidae	S	488	6.03	817	13.22	216	7.75	1025	19.41	699	15.12	249	4.27	1349	21.92	532	10.44	1032	14.58	627	9.28	439	8.16	7473	11.81
Onychiuridae	S	632	7.80	475	7.68	410	14.71	557	10.55	503	10.88	697	11.95	763	12.40	360	7.07	701	9.91	1693	25.06	616	11.45	7407	11.71
Mesostigmata	Pr	522	6.45	576	9.32	484	17.36	605	11.46	548	11.85	600	10.29	695	11.30	440	8.64	718	10.15	540	7.99	647	12.03	6375	10.08
Diptera larvae	S	590	7.28	291	4.71	145	5.20	194	3.67	261	5.65	296	5.08	315	5.12	509	9.99	425	6.01	245	3.63	510	9.48	3781	5.98
Hypogastruridae	S	216	2.67	117	1.89	44	1.58	158	2.99	361	7.81	724	12.42	196	3.19	194	3.81	345	4.87	207	3.06	187	3.48	2749	4.35
Prostigmata	Pr	52	0.64	164	2.65	61	2.19	87	1.65	74	1.60	252	4.32	132	2.15	63	1.24	251	3.55	183	2.71	82	1.52	1401	2.21
Enchytraeidae	S	95	1.17	23	0.37	16	0.57	55	1.04	25	0.54	49	0.84	55	0.89	54	1.06	104	1.47	50	0.74	40	0.74	566	0.89
Tomoceridae	S	10	0.12	137	2.22	13	0.47	29	0.55	2	0.04	29	0.50	63	1.02	64	1.26	116	1.64	50	0.74	22	0.41	535	0.85
Neanridae	Pr	53	0.65	23	0.37	11	0.39	74	1.40	91	1.97	19	0.33	6	0.10	17	0.33	5	0.07	20	0.30	9	0.17	328	0.52
Protura	F	5	0.06	3	0.05	32	1.15	27	0.51	23	0.50	4	0.07	53	0.86	10	0.20	21	0.30	5	0.07	43	0.80	226	0.36
Sminthuridae	S	8	0.10	26	0.42	2	0.07	6	0.11	2	0.04	0	0.00	19	0.31	47	0.92	89	1.26	20	0.30	1	0.02	220	0.35
Entomobryidae	S	7	0.09	15	0.24	6	0.22	57	1.08	0	0.00	33	0.57	20	0.33	11	0.22	58	0.82	10	0.15	2	0.04	219	0.35
Lithobiomorpha	Pr	4	0.05	15	0.24	7	0.25	18	0.34	3	0.06	7	0.12	26	0.42	12	0.24	27	0.38	14	0.21	59	1.10	192	0.30
Stylommatophora	S	16	0.20	8	0.13	3	0.11	26	0.49	27	0.58	2	0.03	14	0.23	10	0.20	58	0.82	24	0.36	3	0.06	191	0.30

Hirudisomatidae	S	1	0.01	26	0.42	12	0.43	7	0.13	1	0.02	10	0.17	15	0.24	3	0.06	11	0.16	10	0.15	16	0.30	112	0.18
Hemiptera larvae	Ph	4	0.05	1	0.02	0	0.00	0	0.00	99	2.14	0	0.00	2	0.03	3	0.06	1	0.01	0	0.00	0	0.00	110	0.17
Formicidae	O	8	0.10	9	0.15	19	0.68	3	0.06	1	0.02	5	0.09	4	0.07	35	0.69	7	0.10	6	0.09	3	0.06	100	0.16
Staphylinidae	S	8	0.10	19	0.31	6	0.22	6	0.11	3	0.06	2	0.03	4	0.07	2	0.04	5	0.07	18	0.27	2	0.04	75	0.12
Ptiliidae	S	12	0.15	1	0.02	13	0.47	11	0.21	7	0.15	0	0.00	4	0.07	9	0.18	4	0.06	2	0.03	7	0.13	70	0.11
Geophilomorpha	Pr	4	0.05	4	0.06	5	0.18	3	0.06	6	0.13	13	0.22	4	0.07	11	0.22	4	0.06	7	0.10	5	0.09	66	0.10
Scutigerellidae	S	3	0.04	2	0.03	5	0.18	10	0.19	7	0.15	11	0.19	7	0.11	4	0.08	1	0.01	6	0.09	5	0.09	61	0.10
Elatcridae larvae	Ph	4	0.05	2	0.03	6	0.22	1	0.02	4	0.09	0	0.00	6	0.10	13	0.26	10	0.14	1	0.01	5	0.09	52	0.08
Chthonidae	Pr	2	0.02	6	0.10	1	0.04	3	0.06	2	0.04	0	0.00	8	0.13	7	0.14	3	0.04	2	0.03	15	0.28	49	0.08
Pselaphidae	Pr	9	0.11	3	0.05	0	0.00	0	0.00	1	0.02	1	0.02	0	0.00	2	0.04	4	0.06	13	0.19	0	0.00	33	0.05
Neelidae	S	0	0.00	0	0.00	1	0.04	6	0.11	5	0.11	0	0.00	4	0.07	6	0.12	2	0.03	5	0.07	0	0.00	29	0.05
Cicadelloidea	Ph	3	0.04	3	0.05	10	0.36	0	0.00	5	0.11	0	0.00	0	0.00	1	0.02	2	0.03	5	0.07	0	0.00	29	0.05
Araneidae	Pr	0	0.00	2	0.03	7	0.25	4	0.08	5	0.11	0	0.00	1	0.02	0	0.00	2	0.03	1	0.01	1	0.02	23	0.04
Curculionidae larvae	Ph	0	0.00	1	0.02	0	0.00	1	0.02	7	0.15	0	0.00	1	0.02	5	0.10	1	0.01	4	0.06	2	0.04	22	0.03
Staphylinidae larvae	S	3	0.04	1	0.02	1	0.04	0	0.00	5	0.11	1	0.02	2	0.03	1	0.02	2	0.03	4	0.06	0	0.00	20	0.03
Coleoptera larvae	Pr	2	0.02	6	0.10	0	0.00	0	0.00	0	0.00	0	0.00	0	0.00	5	0.10	3	0.04	3	0.04	0	0.00	19	0.03
Carabidae larvae	Pr	6	0.07	1	0.02	4	0.14	1	0.02	0	0.00	0	0.00	0	0.00	1	0.02	0	0.00	0	0.00	3	0.06	16	0.03
Lepidoptera larvae	Ph	0	0.00	0	0.00	0	0.00	1	0.02	0	0.00	1	0.02	2	0.03	3	0.06	3	0.04	4	0.06	0	0.00	14	0.02
Scaphidiidae	S	0	0.00	1	0.02	3	0.11	1	0.02	0	0.00	0	0.00	1	0.02	2	0.04	0	0.00	4	0.06	0	0.00	12	0.02
Pauropoda	F	2	0.02	0	0.00	3	0.11	0	0.00	1	0.02	0	0.00	0	0.00	2	0.04	0	0.00	1	0.01	3	0.06	12	0.02
Chrysomelidae larvae	Ph	0	0.00	1	0.02	0	0.00	1	0.02	2	0.04	0	0.00	1	0.02	0	0.00	2	0.03	2	0.03	1	0.02	10	0.02
Silphidae	S	1	0.01	1	0.02	2	0.07	1	0.02	0	0.00	0	0.00	0	0.00	1	0.02	0	0.00	3	0.04	1	0.02	10	0.02
Carabidae	Pr	1	0.01	1	0.02	2	0.07	0	0.00	0	0.00	1	0.02	0	0.00	0	0.00	0	0.00	0	0.00	3	0.06	8	0.01

Lumbricidae	S	0	0.00	1	0.02	4	0.14	2	0.04	1	0.02	0	0.00	0	0.00	0	0.00	0	0.00	0	0.00	0	0.00	8	0.01
Lycosidae	Pr	1	0.01	3	0.05	0	0.00	0	0.00	0	0.00	0	0.00	0	0.00	1	0.02	1	0.01	1	0.01	0	0.00	7	0.01
Cantharidae larvae	Pr	0	0.00	0	0.00	0	0.00	0	0.00	0	0.00	4	0.07	0	0.00	1	0.02	1	0.01	0	0.00	0	0.00	6	0.01
Lampyridae larvae	Pr	1	0.01	0	0.00	0	0.00	0	0.00	0	0.00	0	0.00	1	0.02	1	0.02	1	0.01	2	0.03	0	0.00	6	0.01
Scydmaenidae	Pr	0	0.00	1	0.02	0	0.00	0	0.00	1	0.02	0	0.00	1	0.02	0	0.00	1	0.01	0	0.00	1	0.02	5	0.01
Scarabaeidae	S	1	0.01	1	0.02	0	0.00	0	0.00	1	0.02	0	0.00	0	0.00	0	0.00	1	0.01	0	0.00	0	0.00	4	0.01
Nitidulidae	S	0	0.00	0	0.00	0	0.00	0	0.00	0	0.00	1	0.02	0	0.00	0	0.00	0	0.00	1	0.01	2	0.04	4	0.01
Hebridae	Ph	0	0.00	0	0.00	3	0.11	0	0.00	0	0.00	0	0.00	0	0.00	0	0.00	1	0.01	0	0.00	0	0.00	4	0.01
Mycetophagidae	S	0	0.00	1	0.02	1	0.04	0	0.00	0	0.00	0	0.00	0	0.00	0	0.00	0	0.00	2	0.03	0	0.00	4	0.01
Clubionidae	Pr	0	0.00	2	0.03	0	0.00	0	0.00	0	0.00	0	0.00	0	0.00	0	0.00	0	0.00	0	0.00	1	0.02	3	0.00
Psocoptera	Ph	0	0.00	1	0.02	0	0.00	0	0.00	0	0.00	0	0.00	1	0.02	0	0.00	1	0.01	0	0.00	0	0.00	3	0.00
Paradoxosomatidae	S	1	0.01	0	0.00	0	0.00	0	0.00	0	0.00	0	0.00	0	0.00	1	0.02	1	0.01	0	0.00	0	0.00	3	0.00
Pselaphidae larvae	Pr	1	0.01	0	0.00	0	0.00	1	0.02	0	0.00	1	0.02	0	0.00	0	0.00	0	0.00	0	0.00	0	0.00	3	0.00
Nitidulidae larvae	Pr	0	0.00	0	0.00	2	0.07	0	0.00	0	0.00	0	0.00	0	0.00	0	0.00	0	0.00	0	0.00	0	0.00	2	0.00
Zodariidae	Pr	0	0.00	0	0.00	0	0.00	1	0.02	0	0.00	0	0.00	0	0.00	1	0.02	0	0.00	0	0.00	0	0.00	2	0.00
Saltieidae	Pr	0	0.00	0	0.00	0	0.00	0	0.00	0	0.00	0	0.00	0	0.00	1	0.02	1	0.01	0	0.00	0	0.00	2	0.00
Thomisidae	Pr	0	0.00	0	0.00	0	0.00	0	0.00	0	0.00	0	0.00	0	0.00	1	0.02	1	0.01	0	0.00	0	0.00	2	0.00
Silphidae	S	0	0.00	0	0.00	2	0.07	0	0.00	0	0.00	0	0.00	0	0.00	0	0.00	0	0.00	0	0.00	0	0.00	2	0.00
Hahniidae	Pr	0	0.00	0	0.00	0	0.00	0	0.00	0	0.00	0	0.00	0	0.00	0	0.00	1	0.01	0	0.00	1	0.02	2	0.00
Pyrrhocoridae	Ph	0	0.00	1	0.02	0	0.00	0	0.00	0	0.00	0	0.00	0	0.00	0	0.00	0	0.00	0	0.00	0	0.00	1	0.00
Anthocoridae	Ph	0	0.00	0	0.00	1	0.04	0	0.00	0	0.00	0	0.00	0	0.00	0	0.00	0	0.00	0	0.00	0	0.00	1	0.00
Cantharidae	Pr	0	0.00	0	0.00	0	0.00	0	0.00	0	0.00	0	0.00	0	0.00	1	0.02	0	0.00	0	0.00	0	0.00	1	0.00
Lepidoptera larvae	Ph	0	0.00	0	0.00	1	0.04	0	0.00	0	0.00	0	0.00	0	0.00	0	0.00	0	0.00	0	0.00	0	0.00	1	0.00
Agelenidae	Pr	0	0.00	1	0.02	0	0.00	0	0.00	0	0.00	0	0.00	0	0.00	0	0.00	0	0.00	0	0.00	0	0.00	1	0.00
Coccinellidae	Pr	0	0.00	0	0.00	1	0.04	0	0.00	0	0.00	0	0.00	0	0.00	0	0.00	0	0.00	0	0.00	0	0.00	1	0.00
Gnaphosidae	Pr	0	0.00	0	0.00	0	0.00	0	0.00	0	0.00	0	0.00	0	0.00	0	0.00	0	0.00	0	0.00	1	0.02	1	0.00

Cydnidae	Ph	0	0.00	0	0.00	1	0.04	0	0.00	0	0.00	0	0.00	0	0.00	0	0.00	0	0.00	0	0.00	0	0.00	1	0.00
Scolytidae	Ph	0	0.00	0	0.00	1	0.04	0	0.00	0	0.00	0	0.00	0	0.00	0	0.00	0	0.00	0	0.00	0	0.00	1	0.00
Lampyridae	Pr	0	0.00	0	0.00	0	0.00	1	0.02	0	0.00	0	0.00	0	0.00	0	0.00	0	0.00	0	0.00	0	0.00	1	0.00
Taxa number		37	/	44	/	42	/	35	/	34	/	26	/	34	/	43	/	44	/	39	/	35	/	67	/
Total individual number		8099	/	6181	/	2788	/	5281	/	4623	/	5831	/	6153	/	5094	/	7077	/	6755	/	5378	/	63260	/

October		FM		JM		LG		PS		PA		FM-LG		FM-PS		JM-LG		JM-PS		SF-1		SF-2		Total	
Taxonomic group	Trophic group	Ind	P (%)	Ind	P (%)	Ind	P (%)	Ind	P (%)	Ind	P (%)	Ind	P (%)	Ind	P (%)	Ind	P (%)	Ind	P (%)	Ind	P (%)	Ind	P (%)	Ind	P (%)
Oribatida	S	2055	60.44	3474	58.53	1540	39.28	2302	50.46	1724	34.61	2334	50.71	1972	51.02	3733	57.34	3024	52.89	2697	49.59	2604	52.96	27459	50.99
Onychiuridae	S	236	6.94	488	8.22	780	19.89	820	17.97	557	11.18	683	14.84	359	9.29	507	7.79	506	8.85	855	15.72	527	10.72	6318	11.73
Isotomidae	S	197	5.79	658	11.09	538	13.72	495	10.85	849	17.04	326	7.08	368	9.52	564	8.66	682	11.93	651	11.97	417	8.48	5745	10.67
Mesostigmata	Pr	201	5.91	367	6.18	473	12.06	374	8.20	720	14.45	256	5.56	370	9.57	483	7.42	395	6.91	374	6.88	322	6.55	4335	8.05
Diptera larvae	S	357	10.50	272	4.58	155	3.95	174	3.81	223	4.48	201	4.37	172	4.45	277	4.25	195	3.41	280	5.15	508	10.33	2814	5.23
Prostigmata	Pr	141	4.15	132	2.22	78	1.99	72	1.58	261	5.24	451	9.80	239	6.18	114	1.75	274	4.79	223	4.10	116	2.36	2101	3.90
Hypogastruridae	S	78	2.29	243	4.09	76	1.94	97	2.13	179	3.59	187	4.06	129	3.34	353	5.42	271	4.74	144	2.65	178	3.62	1935	3.59
Enchytraeidae	S	66	1.94	124	2.09	88	2.24	56	1.23	67	1.35	48	1.04	42	1.09	163	2.50	54	0.94	94	1.73	68	1.38	870	1.62
Neanridae	Pr	15	0.44	75	1.26	52	1.33	31	0.68	151	3.03	27	0.59	56	1.45	89	1.37	132	2.31	28	0.51	75	1.53	731	1.36
Entomobryidae	S	7	0.21	13	0.22	25	0.64	52	1.14	32	0.64	8	0.17	28	0.72	28	0.43	51	0.89	16	0.29	5	0.10	265	0.49
Tomoceridae	S	10	0.29	20	0.34	28	0.71	21	0.46	37	0.74	17	0.37	13	0.34	50	0.77	37	0.65	16	0.29	8	0.16	257	0.48

Stylommatophora	S	3	0.09	14	0.24	4	0.10	5	0.11	20	0.40	5	0.11	6	0.16	27	0.41	8	0.14	14	0.26	7	0.14	113	0.21
Scutigerellidae	S	3	0.09	8	0.13	7	0.18	9	0.20	10	0.20	7	0.15	27	0.70	3	0.05	16	0.28	4	0.07	14	0.28	108	0.20
Protura	F	0	0.00	1	0.02	2	0.05	3	0.07	18	0.36	9	0.20	37	0.96	16	0.25	10	0.17	0	0.00	10	0.20	106	0.20
Hemiptera larvae	Ph	2	0.06	0	0.00	24	0.61	0	0.00	71	1.43	2	0.04	3	0.08	0	0.00	2	0.03	1	0.02	0	0.00	105	0.19
Lithobiomorpha	Pr	2	0.06	7	0.12	6	0.15	15	0.33	15	0.30	5	0.11	12	0.31	13	0.20	7	0.12	3	0.06	11	0.22	96	0.18
Hirudisomatidae	S	1	0.03	11	0.19	12	0.31	13	0.28	8	0.16	8	0.17	7	0.18	13	0.20	8	0.14	4	0.07	8	0.16	93	0.17
Chthonidae	Pr	0	0.00	10	0.17	0	0.00	0	0.00	4	0.08	0	0.00	2	0.05	15	0.23	13	0.23	3	0.06	4	0.08	51	0.09
Staphylinidae	S	3	0.09	2	0.03	2	0.05	3	0.07	5	0.10	4	0.09	2	0.05	4	0.06	5	0.09	8	0.15	0	0.00	38	0.07
Formicidae	O	2	0.06	2	0.03	8	0.20	7	0.15	0	0.00	0	0.00	1	0.03	5	0.08	0	0.00	4	0.07	1	0.02	30	0.06
Ptiliidae	S	0	0.00	1	0.02	0	0.00	2	0.04	7	0.14	6	0.13	0	0.00	2	0.03	1	0.02	0	0.00	9	0.18	28	0.05
Araneidae	Pr	2	0.06	1	0.02	2	0.05	3	0.07	4	0.08	0	0.00	1	0.03	8	0.12	0	0.00	2	0.04	2	0.04	25	0.05
Geophilomorpha	Pr	1	0.03	0	0.00	6	0.15	2	0.04	3	0.06	1	0.02	1	0.03	5	0.08	0	0.00	0	0.00	5	0.10	24	0.04
Sminthuridae	S	0	0.00	0	0.00	0	0.00	2	0.04	0	0.00	1	0.02	5	0.13	4	0.06	4	0.07	1	0.02	0	0.00	17	0.03
Thysanoptera	F	0	0.00	1	0.02	0	0.00	0	0.00	0	0.00	3	0.07	1	0.03	3	0.05	1	0.02	6	0.11	1	0.02	16	0.03
Staphylinidae larvae	S	6	0.18	1	0.02	0	0.00	1	0.02	3	0.06	2	0.04	0	0.00	1	0.02	1	0.02	0	0.00	1	0.02	16	0.03
Pselaphidae	Pr	0	0.00	0	0.00	0	0.00	0	0.00	0	0.00	3	0.07	0	0.00	8	0.12	1	0.02	3	0.06	0	0.00	15	0.03
Lepidoptera larvae	Ph	1	0.03	2	0.03	2	0.05	0	0.00	0	0.00	1	0.02	0	0.00	3	0.05	3	0.05	1	0.02	1	0.02	14	0.03
Elatcridae larvae	Ph	0	0.00	1	0.02	1	0.03	0	0.00	2	0.04	1	0.02	4	0.10	1	0.02	1	0.02	1	0.02	0	0.00	12	0.02
Neelidae	S	0	0.00	0	0.00	3	0.08	0	0.00	1	0.02	1	0.02	0	0.00	1	0.02	1	0.02	2	0.04	0	0.00	9	0.02
Nitidulidae larvae	Pr	0	0.00	0	0.00	0	0.00	0	0.00	0	0.00	0	0.00	0	0.00	2	0.03	2	0.03	0	0.00	4	0.08	8	0.01
Scaphidiidae	S	1	0.03	0	0.00	0	0.00	0	0.00	1	0.02	1	0.02	0	0.00	2	0.03	0	0.00	0	0.00	2	0.04	7	0.01
Lampyridae larvae	Pr	0	0.00	1	0.02	0	0.00	0	0.00	0	0.00	2	0.04	1	0.03	1	0.02	2	0.03	0	0.00	0	0.00	7	0.01
Curculionidae	Ph	2	0.06	0	0.00	2	0.05	0	0.00	0	0.00	0	0.00	1	0.03	0	0.00	1	0.02	0	0.00	0	0.00	6	0.01
Chrysomelidae	Ph	0	0.00	0	0.00	1	0.03	0	0.00	0	0.00	0	0.00	2	0.05	1	0.02	2	0.03	0	0.00	0	0.00	6	0.01
Hahniidae	Pr	0	0.00	2	0.03	0	0.00	0	0.00	0	0.00	0	0.00	0	0.00	2	0.03	2	0.03	0	0.00	0	0.00	6	0.01

Lycosidae	Pr	0	0.00	2	0.03	0	0.00	0	0.00	0	0.00	0	0.00	0	0.00	0	0.00	1	0.02	2	0.04	0	0.00	5	0.01
Lumbricidae	S	0	0.00	0	0.00	3	0.08	1	0.02	0	0.00	1	0.02	0	0.00	0	0.00	0	0.00	0	0.00	0	0.00	5	0.01
Curculionidae larvae	Ph	0	0.00	0	0.00	0	0.00	1	0.02	2	0.04	0	0.00	1	0.03	0	0.00	1	0.02	0	0.00	0	0.00	5	0.01
Scolytidae	Ph	4	0.12	0	0.00	0	0.00	0	0.00	0	0.00	0	0.00	0	0.00	0	0.00	1	0.02	0	0.00	0	0.00	5	0.01
Pauropoda	F	0	0.00	0	0.00	1	0.03	1	0.02	1	0.02	0	0.00	0	0.00	1	0.02	0	0.00	1	0.02	0	0.00	5	0.01
Carabidae	Pr	0	0.00	1	0.02	0	0.00	0	0.00	0	0.00	0	0.00	1	0.03	0	0.00	1	0.02	0	0.00	1	0.02	4	0.01
Scarabaeidae	S	0	0.00	0	0.00	0	0.00	0	0.00	0	0.00	0	0.00	0	0.00	0	0.00	0	0.00	0	0.00	3	0.06	3	0.01
Tenebrionidae	Pr	0	0.00	0	0.00	0	0.00	0	0.00	3	0.06	0	0.00	0	0.00	0	0.00	0	0.00	0	0.00	0	0.00	3	0.01
Clubionidae	Pr	0	0.00	0	0.00	1	0.03	0	0.00	0	0.00	0	0.00	0	0.00	0	0.00	0	0.00	1	0.02	0	0.00	2	0.00
Liocranidae	Pr	1	0.03	0	0.00	1	0.03	0	0.00	0	0.00	0	0.00	0	0.00	0	0.00	0	0.00	0	0.00	0	0.00	2	0.00
Cantharidae larvae	Pr	0	0.00	0	0.00	0	0.00	0	0.00	1	0.02	0	0.00	1	0.03	0	0.00	0	0.00	0	0.00	0	0.00	2	0.00
Paradoxosomatidae	S	1	0.03	0	0.00	0	0.00	0	0.00	0	0.00	0	0.00	0	0.00	0	0.00	0	0.00	0	0.00	1	0.02	2	0.00
Chelonariidae	S	0	0.00	0	0.00	0	0.00	0	0.00	0	0.00	0	0.00	0	0.00	2	0.03	0	0.00	0	0.00	0	0.00	2	0.00
Coleoptera larvae	Pr	0	0.00	0	0.00	0	0.00	0	0.00	0	0.00	2	0.04	0	0.00	0	0.00	0	0.00	0	0.00	0	0.00	2	0.00
Thomisidae	Pr	0	0.00	0	0.00	0	0.00	0	0.00	0	0.00	0	0.00	0	0.00	1	0.02	1	0.02	0	0.00	0	0.00	2	0.00
Cicadelloidea	Ph	1	0.03	0	0.00	0	0.00	0	0.00	0	0.00	0	0.00	0	0.00	0	0.00	0	0.00	0	0.00	1	0.02	2	0.00
Cleridae larvae	Pr	0	0.00	1	0.02	0	0.00	0	0.00	0	0.00	0	0.00	0	0.00	0	0.00	0	0.00	0	0.00	0	0.00	1	0.00
Silvanidae	Ph	0	0.00	0	0.00	0	0.00	0	0.00	0	0.00	0	0.00	0	0.00	0	0.00	1	0.02	0	0.00	0	0.00	1	0.00
Elatcridae	Ph	0	0.00	0	0.00	0	0.00	0	0.00	0	0.00	0	0.00	1	0.03	0	0.00	0	0.00	0	0.00	0	0.00	1	0.00
Zoridae	Pr	0	0.00	0	0.00	0	0.00	0	0.00	0	0.00	0	0.00	0	0.00	1	0.02	0	0.00	0	0.00	0	0.00	1	0.00
Lepidoptera larvae	Ph	0	0.00	0	0.00	0	0.00	0	0.00	0	0.00	0	0.00	0	0.00	0	0.00	0	0.00	0	0.00	1	0.02	1	0.00
Nitidulidae	S	0	0.00	0	0.00	0	0.00	0	0.00	1	0.02	0	0.00	0	0.00	0	0.00	0	0.00	0	0.00	0	0.00	1	0.00
Zodariidae	Pr	0	0.00	0	0.00	0	0.00	0	0.00	0	0.00	0	0.00	0	0.00	1	0.02	0	0.00	0	0.00	0	0.00	1	0.00
Diplura	Pr	0	0.00	0	0.00	0	0.00	0	0.00	0	0.00	0	0.00	0	0.00	1	0.02	0	0.00	0	0.00	0	0.00	1	0.00
Hydrophilidae	S	0	0.00	0	0.00	0	0.00	0	0.00	0	0.00	0	0.00	0	0.00	1	0.02	0	0.00	0	0.00	0	0.00	1	0.00

Saltieidae	Pr	0	0.00	0	0.00	0	0.00	0	0.00	0	0.00	0	0.00	0	0.00	0	0.00	0	0.00	0	0.00	1	0.02	1	0.00
Endomychidae	S	0	0.00	0	0.00	0	0.00	0	0.00	0	0.00	0	0.00	0	0.00	1	0.02	0	0.00	0	0.00	0	0.00	1	0.00
Mycetophagidae	S	0	0.00	0	0.00	0	0.00	0	0.00	1	0.02	0	0.00	0	0.00	0	0.00	0	0.00	0	0.00	0	0.00	1	0.00
Silphidae	S	1	0.03	0	0.00	0	0.00	0	0.00	0	0.00	0	0.00	0	0.00	0	0.00	0	0.00	0	0.00	0	0.00	1	0.00
Lygaeidae	Ph	0	0.00	0	0.00	0	0.00	0	0.00	0	0.00	0	0.00	0	0.00	0	0.00	0	0.00	0	0.00	1	0.02	1	0.00
Taxa number		29	/	30	/	30	/	26	/	32	/	31	/	32	/	42	/	38	/	29	/	33	/	66	/
Total individual number		3400	/	5935	/	3921	/	4562	/	4981	/	4603	/	3865	/	6510	/	5718	/	5439	/	4917	/	53851	/

Note: Ind: individual number; P: Percent. S: saprophagous, Pr: predaceous, O: omnivorous, Ph: phytophagous, F: fungivore. FM, JM, LG, PS, PA, FM-LG, FM-PS, JM-LG, JM-PS for plantation forest stands composed by *Fraxinus mandshurica, Juglans mandshurica, Larix gmelinii, Pinus koraiensis, Picea koraiensis, Fraxinus mandshurica* and *Larix gmelinii, Fraxinus mandshurica* and *Pinus koraiensis, Juglans mandshurica* and *Larix gmelinii, Juglans mandshurica* and *Pinus koraiensis*， respectively. SF-1 and SF-2 for second forest stands composed by *Fraxinus mandshurica, Betula platyphylla, Ulmus davidiana*, *Juglans mandshurica, Quercus mongolica*, *Quercus mongolica*, *Acer mono,Tilia.*

	Shannon(understory)	Simpson(understory)	richness(species·m^{-2})	coverage(%)	LT/cm	Tem ℃	Mois (%)
FM	1.46±0.24a	0.65±0.09a	7.80±1.36a	86.60±8.11a	1.70±0.12d	18.51±0.36ab	34.25±4.16a
JM	0.60±0.22ab	0.30±0.11ab	4.20±1.28abc	26.80±13.88bc	2.40±0.24bcd	17.70±0.22bc	30.93±3.82ab
LG	1.30±0.14a	0.61±0.05a	7.80±0.92a	75.40±8.1a	4.20±0.68b	17.09±0.22c	24.75±1.24ab
PS	0.64±0.24ab	0.35±0.13ab	3.60±0.68abc	21.50±7.99bc	3.00±0.42bcd	17.28±0.06c	27.83±1.52ab
PA	0b	0b	0d	0c	5.70±0.64a	16.37±0.24d	22.80±0.30b
FM-LG	1.25±0.15a	0.46±0.14a	6.60±0.40abc	84.40±5.85a	2.00±0.27cd	18.27±0.40ab	32.78±1.86a
FM-PS	0.72±0.20ab	0.41±0.11a	2.80±0.97c	12.40±5.09bc	3.50±0.45bcd	18.66±0.15a	12.66±0.49c
JM-LG	0.70±0.28ab	0.34±0.13ab	4.00±0.77abc	54.60±9.08ab	3.90±0.71bc	18.31±0.08ab	28.85±1.05ab

Table S2. Understory characteristics, litter thickness, soil temperature and moisture content (mean ± SD) of the 11 stands.

JM-PS	1.01±0.20a	0.44±0.09a	4.00±0.84abc	30.00±6.83bc	2.30±0.20bcd	18.24±0.08ab	28.41±2.67ab
SF-1	1.26±0.21a	0.60±0.07a	7.40±0.93ab	54.40±15.51ab	2.40±0.29bcd	17.76±0.11bc	30.13±2.00ab
SF-2	1.13±0.09a	0.63±0.04a	4.00±0.55abc	37.20±14.96bc	3.00±0.45bcd	17.70±0.11bc	26.49±1.57ab
F	4.783	3.901	7.746	9.115	6.669	10.473	6.996
P	P<0.000***	P=0.001***	P<0.000***	P<0.000***	P<0.000***	P<0.000***	P<0.000***

Note: Lowercase letters indicate significant differences between stands. *, ** and *** indicate significant differences at the $P < 0.05$ level, $P < 0.01$ level and $P < 0.001$ level, respectively. FM, JM, LG, PS, PA, FM-LG, FM-PS, JM-LG, JM-PS for plantation forest stands composed by *Fraxinus mandshurica, Juglans mandshurica, Larix gmelinii, Pinus koraiensis, Picea koraiensis, Fraxinus mandshurica* and *Larix gmelinii, Fraxinus mandshurica* and *Pinus koraiensis, Juglans mandshurica* and *Larix gmelinii, Juglans mandshurica* and *Pinus koraiensis*，respectively. SF-1 and SF-2 for second forest stands composed by *Fraxinus mandshurica, Betula platyphylla, Ulmus davidiana*, *Juglans mandshurica, Quercus mongolica*, *Quercus mongolica*, *Acer mono,Tilia.*

Table S3. Soil chemical properties (mean ± SD) of the 11 stands.

	pH (H_2O)	TN ($mg·g^{-1}$)	C/N ratio	TP ($mg·g^{-1}$)	TK ($mg·g^{-1}$)	AP ($mg·kg^{-1}$)	AK ($mg·kg^{-1}$)	NO^{3-} ($mg·kg^{-1}$)	NH^{4+} ($mg·kg^{-1}$)	OM ($mg·g^{-1}$)
FM	5.53±0.15b	1.81±0.23a	4.64±0.68b	4.04±0.45b	37.55±2.79	24.52±2.88d	186.35±14.23b	6.60±0.75cd	24.40±2.06a	99.29±7.56bc
JM	5.48±0.08b	0.77±0.06b	10.64±0.14a	3.80±0.59b	45.09±4.17	36.93±4.67cd	275.19±21.45ab	4.71±0.88d	20.31±1.42ab	114.3±10.63bc
LG	5.50±0.04b	0.86±0.03b	10.34±0.26a	3.08±0.18b	37.65±2.09	36.57±4.21cd	210.13±11.85ab	6.42±0.64cd	18.64±0.89b	119.23±3.90b
PS	5.63±0.05b	0.62±0.04b	11.06±0.31a	2.67±0.23b	37.04±2.20	36.68±2.57cd	183.53±8.84b	14.02±1.36ab	19.18±1.22ab	110.52±7.28bc
PA	5.61±0.07b	0.68±0.04b	11.00±0.11a	3.16±0.13b	37.51±5.08	30.57±5.84cd	256.26±27.14ab	14.30±1.13ab	18.62±1.12b	156.56±13.64a
FM-LG	5.50±0.06b	0.99±0.02b	11.26±0.28a	4.15±0.2b	37.97±1.89	28.58±2.87cd	225.48±15.64ab	15.64±1.24a	24.94±2.03a	152.58±7.92a
FM-PS	5.98±0.08a	0.82±0.11b	8.58±0.42a	3.10±0.21b	36.72±1.62	46.36±1.48c	185.15±6.47b	14.84±1.01ab	22.06±1.62ab	105.67±4.34bc
JM-LG	5.50±0.06b	0.56±0.07b	11.00±0.34a	2.80±0.24b	31.86±1.66	46.04±4.16c	192.20±11.5b	9.99±1.06bc	21.92±1.11ab	79.69±5.08c

JM-PS	5.76±0.11ab	1.30±0.60ab	10.08±1.75a	4.10±0.28b	44.06±2.30	36.10±3.15cd	275.09±33.67ab	16.06±1.84a	20.03±2.02ab	125.53±11.27ab
SF-1	5.85±0.09ab	0.83±0.04b	11.23±0.25a	3.61±0.59b	35.01±1.59	87.81±4.27a	290.00±29.14a	16.48±1.79a	23.01±1.57ab	153.64±10.8a
SF-2	5.99±0.07a	0.51±0.01b	11.43±0.20a	7.29±0.71a	38.35±1.53	72.74±8.15b	268.45±22.64ab	11.54±1.45ab	18.07±1.03b	109.9±3.73bc
F	5.805	3.495	10.357	10.307	1.898	19.68	4.304	11.778	2.498	8.417
P	P<0.000***	P=0.002**	P<0.000***	P<0.000***	P=0.071	P<0.000***	P<0.000***	P<0.000***	P=0.018**	P<0.000***

Note: Lowercase letters indicate significant differences between stands. *, ** and *** indicate significant differences at the $P < 0.05$ level, $P < 0.01$ level and $P < 0.001$ level, respectively. FM, JM, LG, PS, PA, FM-LG, FM-PS, JM-LG, JM-PS for plantation forest stands composed by *Fraxinus mandshurica, Juglans mandshurica, Larix gmelinii, Pinus koraiensis, Picea koraiensis, Fraxinus mandshurica* and *Larix gmelinii, Fraxinus mandshurica* and *Pinus koraiensis, Juglans mandshurica* and *Larix gmelinii, Juglans mandshurica* and *Pinus koraiensis*， respectively. SF-1 and SF-2 for second forest stands composed by *Fraxinus mandshurica, Betula platyphylla, Ulmus davidiana*, *Juglans mandshurica, Quercus mongolica*, *Quercus mongolica*, *Acer mono,Tilia.*

Table S4. Results of redundancy analysis model of soil meso- and micro-fauna community variation using biological and abiotic environmental variables, determined by forward selection procedure with unrestricted permutation tests.

Factor	RDA1	RDA2	R^2	*p* value
Shannon index	0.2027	0.9792	0.1104	0.0450*
Simpson index	0.3572	0.9340	0.0598	0.2089
Plant coverage	-0.1020	0.9948	0.5803	0.0005**
Litter thickness	0.9357	-0.3528	0.2194	0.0020**
Soil temperature	-0.8593	0.5115	0.4865	0.0005**
Soil moisture	-0.2264	0.9740	0.1044	0.0640

pH	-0.7380	-0.6748	0.2303	0.0020**
C	-0.6988	-0.7153	0.1804	0.0085**
N	-0.5568	0.8307	0.1836	0.0135*
C/N	0.1491	-0.9888	0.2034	0.0035**
P	-0.7280	0.6856	0.0409	0.3563
K	-0.5205	-0.8539	0.0095	0.7966
AP	-0.9298	0.3682	0.0723	0.1629
AK	-0.9966	0.0829	0.0827	0.1159
NO_3^-	-0.0686	-0.9976	0.4166	0.0005**
NH_4^+	-0.9979	0.0646	0.0372	0.3738
OM	0.7588	-0.6514	0.1977	0.0045**

Note: * and ** indicate significant differences at the $P < 0.05$ level and $P < 0.01$ level, respectively.
